# Modeling intra-mosquito dynamics of Zika virus and its dose-dependence confirms the low epidemic potential of *Aedes albopictus*

**DOI:** 10.1371/journal.ppat.1009068

**Published:** 2020-12-31

**Authors:** Sebastian Lequime, Jean-Sébastien Dehecq, Séverine Matheus, Franck de Laval, Lionel Almeras, Sébastien Briolant, Albin Fontaine

**Affiliations:** 1 Cluster of Microbial Ecology, Groningen Institute for Evolutionary Life Sciences, University of Groningen, Groningen, The Netherlands; 2 KU Leuven Department of Microbiology, Immunology and Transplantation, Rega Institute, Laboratory of Clinical and Epidemiological Virology, Leuven, Belgium; 3 French Ministry of Health, Agence Régionale de Santé de La Réunion, Vector control Unit, La Reunion Island, Saint-Denis, France; 4 Laboratory of Virology, National Reference Center for Arboviruses, Institut Pasteur, Guyane Française, Cayenne, France; 5 Environment and infections risks unit, Institut Pasteur, Paris, France; 6 SSA, Service de Santé des Armées, CESPA, Centre d’épidémiologie et de santé publique des armées, Marseille, France; 7 Aix Marseille Univ, INSERM, IRD, SESSTIM, Sciences Economiques & Sociales de la Santé & Traitement de l’Information Médicale, Marseille, France; 8 Unité Parasitologie et Entomologie, Département Microbiologie et maladies infectieuses, Institut de Recherche Biomédicale des Armées (IRBA), Marseille, France; 9 Aix Marseille Univ, IRD, SSA, AP-HM, UMR Vecteurs–Infections Tropicales et Méditerranéennes (VITROME), Marseille, France; 10 IHU Méditerranée Infection, Marseille, France; Pennsylvania State University, UNITED STATES

## Abstract

Originating from African forests, Zika virus (ZIKV) has now emerged worldwide in urbanized areas, mainly transmitted by *Aedes aegypti* mosquitoes. Although *Aedes albopictus* can transmit ZIKV experimentally and was suspected to be a ZIKV vector in Central Africa, the potential of this species to sustain virus transmission was yet to be uncovered until the end of 2019, when several autochthonous transmissions of the virus vectored by *Ae*. *albopictus* occurred in France. Aside from these few locally acquired ZIKV infections, most territories colonized by *Ae*. *albopictus* have been spared so far. The risk level of ZIKV emergence in these areas remains however an open question. To assess *Ae*. *albopictus*’ vector potential for ZIKV and identify key virus outbreak predictors, we built a complete framework using the complementary combination of (i) dose-dependent experimental *Ae*. *albopictus* exposure to ZIKV followed by time-dependent assessment of infection and systemic infection rates, (ii) modeling of intra-human ZIKV viremia dynamics, and (iii) *in silico* epidemiological simulations using an Agent-Based Model. The highest risk of transmission occurred during the pre-symptomatic stage of the disease, at the peak of viremia. At this dose, mosquito infection probability was estimated to be 20%, and 21 days were required to reach the median systemic infection rates. Mosquito population origin, either temperate or tropical, had no impact on infection rates or intra-host virus dynamic. Despite these unfavorable characteristics for transmission, *Ae*. *albopictus* was still able to trigger and yield large outbreaks in a simulated environment in the presence of sufficiently high mosquito biting rates. Our results reveal a low but existing epidemic potential of *Ae*. *albopictus* for ZIKV, that might explain the absence of large scale ZIKV epidemics so far in territories occupied only by *Ae*. *albopictus*. They nevertheless support active surveillance and eradication programs in these territories to maintain the risk of emergence to a low level.

## Author summary

Zika virus (ZIKV) has emerged worldwide and triggered large outbreaks in human populations. While the yellow fever mosquito *Aedes aegypti* is considered the primary vector of ZIKV, the Asian tiger mosquito *Aedes albopictus* has been shown experimentally to transmit the virus and has been involved in a few autochthonous transmission in France in 2019. Here, we provide a comprehensive study on the ability of *Ae*. *albopictus* mosquitoes to transmit ZIKV by considering the within-host dynamics of ZIKV infection in humans and its impact on both mosquito infection probability and time to mosquito infectiousness. These empirical data were then leveraged by *in silico* simulations to embed them into their epidemiological context. Our study reveals a low but existing epidemic potential of *Ae*. *albopictus* for ZIKV, whatever their tropical or temperate origins. We identified mosquito density as a predictor for ZIKV outbreak occurrence when vectored by *Ae*. *albopictus*. Our findings help to explain the absence of large scale ZIKV epidemics in territories occupied by *Ae*. *albopictus* but call for active surveillance and eradication programs to maintain the risk of emergence to a low level.

## Introduction

Several mosquito-borne viruses have spilled from their primary enzootic cycles in tropical primary forests to emerge worldwide in transmission cycles involving humans and mosquitoes highly adapted to urban environments. While the yellow fever mosquito *Aedes aegypti* is considered as the primary vector of viruses affecting human health, the Asian tiger mosquito *Aedes albopictus* might progressively take the lead because of its outstanding invasive capacity [1] and its ability to transmit numerous virus species experimentally [2]. Originating from South-East Asia, *Ae*. *albopictus* has indeed invaded the world and is now present in all inhabited continents, including temperate Europe, owing to its potential to endure harsh winter conditions [3,4]. The species is also displacing *Ae*. *aegypti* populations in areas where both co-exist due to competitive advantages, notably at the larval stage [5–7].

Zika virus (ZIKV) is the lastest globally-emerging mosquito-borne virus that can be maintained in urban cycles that only involve human-mosquito transmissions. ZIKV is an RNA virus from the *Flavivirus* genus (Flaviviridae), which includes other human pathogens such as yellow fever virus (YFV) or dengue viruses (DENV). First isolated in 1947 from a rhesus monkey in the Zika forest in Uganda, ZIKV has concomitantly emerged in 2007 in Gabon [8] and in the Federated States of Micronesia where it triggered a significant outbreak on Yap Island [9]. The virus then spread through the South Pacific islands from 2013 to 2014 [10,11] before it emerged in north-eastern Brazil in 2015 [12], where it started a large outbreak hitting a total of 50 territories and countries in the Americas [13]. Most ZIKV infections are asymptomatic or cause self-limiting febrile sickness but are sometimes associated with complications, including Guillain-Barré syndrome or congenital microcephaly or fetal losses in women infected during pregnancy [14].

*Ae*. *albopictus* mosquitoes are known to be competent vectors of several major arthropod-borne viruses (arboviruses) [2]. They have been incriminated in the transmission of chikungunya virus (CHIKV) and DENV viruses in recent outbreaks in La Réunion island, a French oversea territory in the Indian Ocean [15,16], as well as autochthonous transmission cases or outbreaks in Europe [17–20]. European French populations of *Ae*. *albopictus* mosquitoes were shown experimentally to transmit DENV and CHIKV as efficiently as the typical tropical vector *Ae*. *aegypti* [21]. The presence of barriers preventing systemic ZIKV infection (virus dissemination from the midgut to secondary tissues) or transmission were repeatedly reported for *Ae*. *albopictus* [22–26] and suggested a limited risk of Zika virus transmission in Europe or La Réunion island [22,24]. However, *Ae*. *albopictus* was strongly suspected to be the main vector of ZIKV in Central Africa [8], and in three cases of autochthonous ZIKV transmissions without evidence of sexual transmission that occurred at the end of 2019 in South Metropolitan France where *Ae*. *albopictus*, but not *Ae*. *aegypti*, is well established [27]. Temperate regions invaded by *Ae*. *albopictus* are of high risk of importation of mosquito-borne viruses. Indeed, because of extensive trade and tourism, these are highly connected to regions repeatedly hit by mosquito-borne viruses.

The intrinsic ability of a mosquito to be infected and subsequently transmit a virus, termed vector competence, is strongly influenced by numerous factors, including the infectious oral dose received by mosquitoes and the time post-exposure [28]. In the present study, we assessed the susceptibility of *Ae*. *albopictus* populations from Marseille (Metropolitan France) and La Réunion island (French overseas territory in the Indian Ocean) for ZIKV infection and compared their intra-host systemic infection dynamics. We also experimentally investigated how intra-human ZIKV load dynamics influence mosquito infection probability and systemic infection dynamics. We then implemented *in silico* epidemiological simulations to leverage these empirically acquired data into a realistic framework to assess the potential of *Ae*. *albopictus* as a vector of ZIKV and to identify strong virus outbreak predictors.

## Results

### Modeling of ZIKV loads dynamic in human venous blood

**Fig 1:**
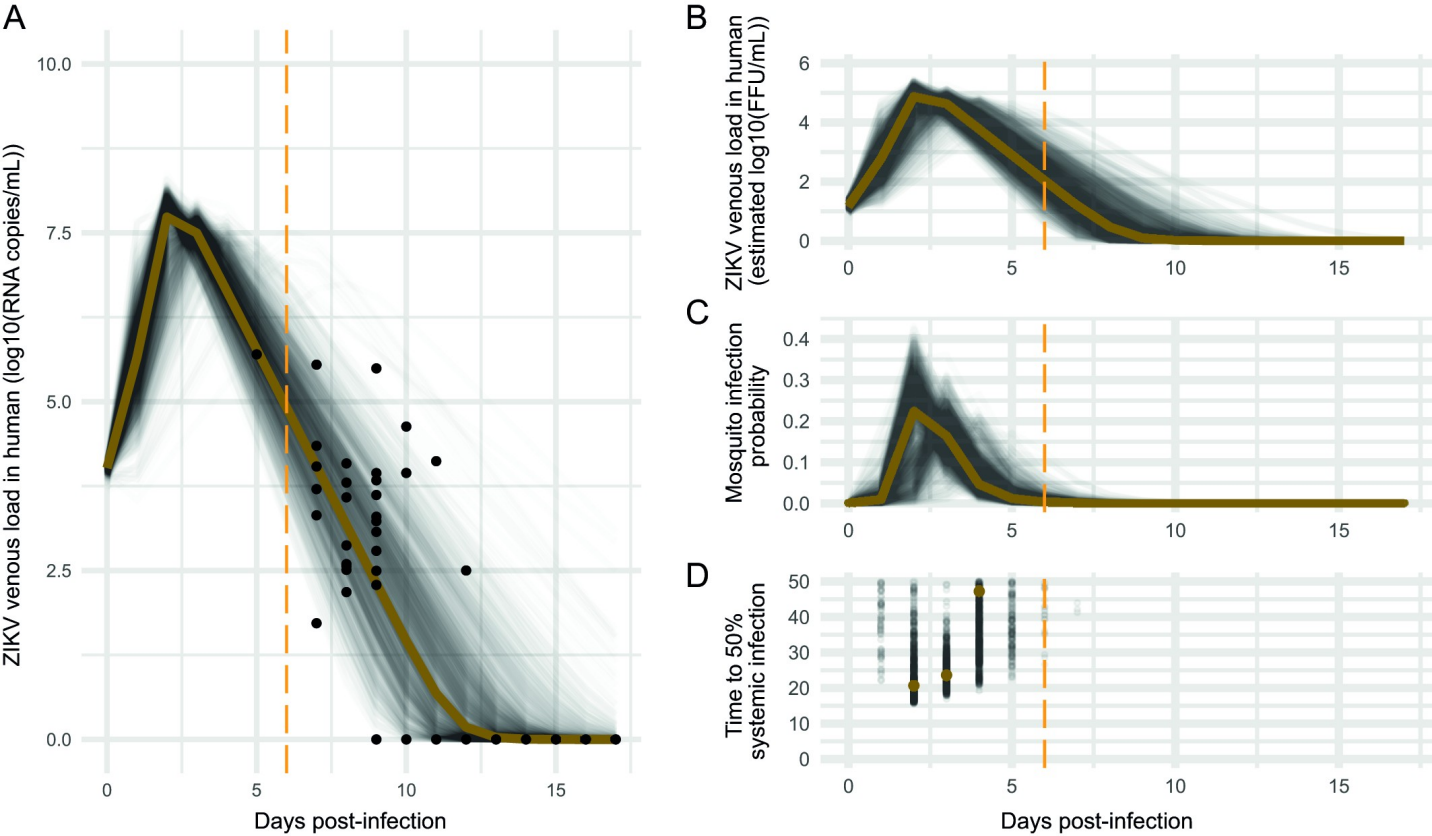
Intra-human ZIKV loads dynamic modelisation and its direct impact on mosquito infection and systemic infection dynamic. A- Estimated ZIKV viremia (RNA copies/mL) dynamic post-infection in humans. Bayesian inference was used to fit a target-cell limited model with a latent phase on viral genomic loads dynamics averaged across patients using parameter values estimates from non-human primates as strong informative priors. The golden line represents model prediction using mean fit parameter values. ZIKV loads in patients are represented with dots. Thin black lines represent model prediction for 1,000 independent individuals. For each individual, each parameter value was drawn from a normal distribution in the form *N(μ =* mean parameter estimate, *σ2 = μ/6)*, with 6 being a scaling factor. The scaling factor was determined arbitrarily so that the distribution of predicted viremia dynamics cover patient data. B- Modelisation of ZIKV venous load (FFU/mL) dynamic post-infection in humans. The golden line represents model prediction using mean fit parameter values, as determined in A. Thin black lines represent model prediction for 1,000 independent individuals. These viremia dynamics were directly inferred from RNA copies/mL displayed in (A) by applying a conversion factor. C- Mosquito infection probability dynamic directly inferred from the intra-human ZIKV viremia in (B). Logistic regression was used to model and predict the virus dose effect on the probability of mosquito infection based on our experimental dose-response data. D- Median time to systemic infection dynamic as a function of the intra-human ZIKV viremia in (B). Median times to reach 50% systemic infection (virus dissemination) were estimated for non-null mosquito infection probabilities. The Y-axis was limited to 50 days, a time that was estimated to largely exceed the mosquito life expectancy in nature. The vertical orange dashed line represents the time to symptom onset in humans.

We aimed to provide a realistic Zika viremia dynamic in human post-infection based on viral loads in sera collected from human venous blood. ZIKV loads in venous sera from symptomatic patients ranged from 0 to 3.2 × 10^5^ RNA copies/mL in the post-symptomatic stage of the infection. One serum had an estimated virus load of 5 × 10^5^ RNA copies/mL on the day before symptom onsets. ZIKV load dropped by 2 log points (*i*.*e*., 5 × 10^3^ RNA copies/mL) for this patient one day after symptom onsets. We fitted a target-cell limited model [29] with a latent phase on viral genomic loads dynamics averaged across patients using parameter values estimates from experimental assays in non-human primates [29] as strong informative priors. The numerical integration of the model is plotted in Fig 1A (gold line) with ZIKV loads from human venous blood as dots. Prior distributions had a considerable influence on the posterior distribution for parameters *T0* (initial uninfected target cells concentration, 45% prior posterior overlap (PPO)), *V0* (initial concentration of free virus particles, 43% PPO), *k* (rate of cell transition from a non-productive sate to a virus productive state, 35% PPO) and *p* (free virus releasing rate by productively infected cells, 45% PPO). Our data were not informative enough to overcome the influence of the prior distribution for these parameters (S1 Fig).

Our modeled ZIKV plasma loads dynamic in humans was characterized by a period of exponential growth that reaches a peak of 5.4 × 10^7^ ZIKV RNA copies/mL at day 2 post-infection (equivalent to an estimated 7.2 × 10^4^ FFU/mL), followed by a period of exponential decline until the virus becomes undetectable around 12 days post-infection (Fig 1A). An infectious inoculum with an estimated mean of 10,415 ZIKV RNA copies per mL (posterior sample quantiles (psq) 2.5%: 10,015; 97.5%: 11,184 RNA copies/mL) in contact with an estimated mean of 1.1 × 10^5^ (psq 2.5%: 1 × 10^5^, 97.5%: 1.2 × 10^5^) uninfected target cells per mL initiated the ZIKV infection. The average transition time estimate from the latent phase to the virus productive phase was 2.3 hours (h) (psq 2.5%: 1.5 h; 97.5%: 3.8 h). Productively infected cells release 26,579 (psq 2.5%: 25,053; 97.5%: 29,901) free virus per cell per day and have an average lifespan of 12h. The number of secondary cells infected by ZIKV production from one infected cell in a population of target cells was estimated to 7.6.

### Infection rates and systemic infection dynamics across mosquito populations

**Fig 2:**
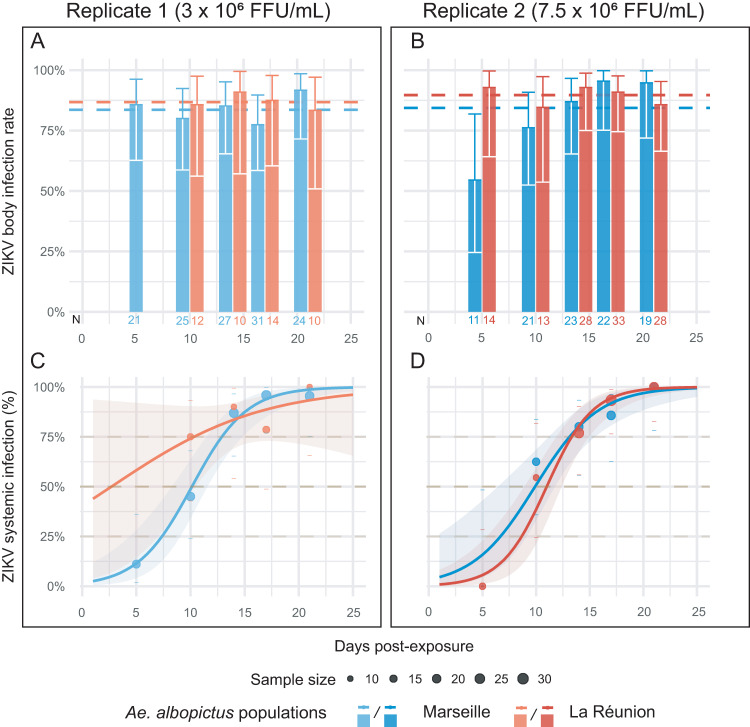
**Body infection prevalence (A, B) and systemic infection dynamics (C, D) for two *Ae*. *albopictus* mosquito populations exposed the same isolate of ZIKV.** Pannel A, C and B, D represent two experiment duplicates from which the infectious blood meal was tittered at 3 × 10^6^ FFU/mL (A, C) and 7.5 × 10^6^ FFU/mL (B, D). Percentages of body infections over time post-ZIKV exposure are represented for the Marseille (Metropolitan France) population (blue color) and La Réunion (Overseas France) population (red color) in both replicates. The sample size is indicated below each bar. Prevalences averaged across time points are represented with a dashed line for each mosquito population. 95% confidence intervals are indicated with the error bars. Cumulative prevalence of systemic (disseminated) infections over time post ZIKV exposure are represented as points for the Metropolitan France population (blue color) and Overseas France population (red color) (C, D). Dashes represent the 95% confidence intervals of prevalences. Dots size indicates the number of samples. The fitted values obtained with a logistic model are represented for each population by a line.

We aimed to expose *Ae*. *albopictus* mosquito populations orally to ZIKV at a titre of 2 × 10^6^ FFU/mL. This ZIKV load has a 2 log point drop from the highest ZIKV loads in human venous blood (expressed in ZIKV RNA copies per mL) that were inferred from our intra-human dynamics analysis. These virus titers also lie between median infection doses that were reported for *Ae*. *albopictus* (*i*.*e*., from 6.1 to 6.8 log_10_ FFU/mL) [25,26]. A total of 227 and 175 engorged female mosquitoes from Marseille and La Réunion populations, respectively, were experimentally exposed to one ZIKV isolate. The virus was isolated from the plasma of a traveler returning from Suriname in 2016 that belongs to the Asian lineage. Two ZIKV experimental exposure assays replicates were performed with infectious blood meals titered at 7.5 × 10^6^ FFU/mL (98 and 120 engorged mosquitoes for Marseille and La Réunion populations, respectively) and 3 × 10^6^ FFU/mL (129 and 55 engorged mosquitoes for Marseille and La Réunion populations, respectively). Because each assay was performed with a single dose, the virus dose effect was here confounded with the experiment effect, but infection and systemic infection rates can be directly compared between mosquito populations in each replicate. High body infection prevalences were obtained for both populations, independently of the time post-exposure (Fig 2A and 2B). Averaged over time, mean body infection prevalences were 84% for the Marseille population for each replicate and over 86% for the La Réunion population. A full factorial logistic regression model revealed no statistically significant difference for body infection prevalences across mosquito populations and replicates. However, there was a statistically significant interaction effect between time post-virus exposure and the replicate (analysis of deviance, p-value = 0.048), which was driven by a relatively low body prevalence at 5 days post-virus exposure for mosquitoes from the Marseille population at 7.5 × 10^6^ FFU/mL.

Systemic infection prevalences were measured by counting the number of mosquitoes with infected heads over the number of mosquitoes with an infected body (Fig 2C and 2D). Systemic infection prevalences were significantly influenced by the time post-exposure (analysis of deviance, p-value < 2 ×10^−16^) but not by mosquito population or replicate. The intra-host dynamic of systemic ZIKV infection was inferred in all combinations of mosquito population and virus doses by fitting a logistic model. The model failed to provide relevant estimates for the La Réunion population at the lowest virus dose due to a lack of samples at early times post-virus exposure. However, systemic infection prevalences saturated at 100% for both mosquito populations and replicates. The estimated time required to reach 50% of systemic infections was 10.1 (Standard Error: 0.6) and 10 (SE: 0.8) days for the Marseille and the La Réunion populations, respectively, independently of the replicate.

### Mosquito infection rates as a function of the oral ZIKV dose

**Fig 3:**
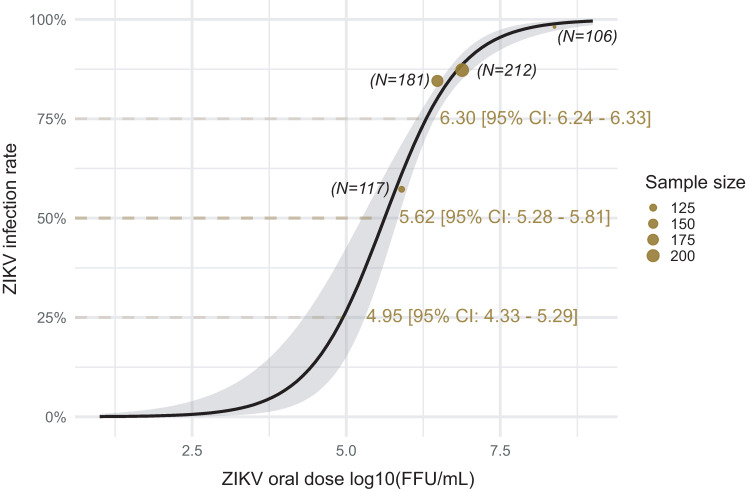
Dose response curve representing *Ae*. *albopictus* mosquito infection rate as a function of ZIKV oral dose. Golden dots correspond to infection rates observed on *Ae*. *albopictus* mosquitoes at each infectious blood-meal titer. Dots size is proportional to the number of samples. The black line corresponds to fit values obtained by fitting a logistic model to the data. The grey ribbon indicates the 95% confidence intervals. The dose to infect 50% of the ZIKV exposed mosquitoes (ID-50), the 25^th^ and 75^th^ percentile are represented with their 95% confidence intervals.

*Ae*. *albopictus* mosquitoes from the La Réunion population were orally challenged with two additional doses of ZIKV (8 × 10^5^ FFU/mL and 2.37 × 10^8^ FFU/mL) in order to assess the effect of the virus dose on mosquito infection probabilities. Mosquitoes were held at a standardized 28°C temperature during the rearing and experimental procedure. This dose-response analysis was performed independently of the mosquito populations (*i*.*e*., both populations were considered as a single one) because no difference was previously revealed for both infection and systemic infection dynamics across populations. Mosquito infection rates increased as a function of the oral ZIKV dose (S2 Fig) without a significant effect of the time post-infection (analysis of deviance). Independently of the time post-infection, the observed prevalence of mosquito infection were 57.3%, 84.5%, 87.3% and 98% for the 8 × 10^5^ FFU/mL, 3 × 10^6^ FFU/mL, 7.5 × 10^6^ FFU/mL and 2.37 × 10^8^ FFU/mL infectious blood meal concentrations, respectively. A logistic model was fit to the data by considering the blood meal virus titer as a unique explanatory variable. The infectious dose required to infect 50% (ID50) of *Ae*. *albopictus* mosquitoes was 4.2 × 10^5^ FFU/mL (95% CI: 1.9 × 10^5^ FFU/mL—6.5 × 10^5^ FFU/mL) (Fig 3). One-quarter of the mosquitoes were infected at 9 × 10^4^ FFU/mL, and 95% of mosquitoes were infected at 2 × 10^6^ FFU/mL.

### Intra-mosquito ZIKV systemic infection dynamics as a function of the oral ZIKV dose

**Fig 4:**
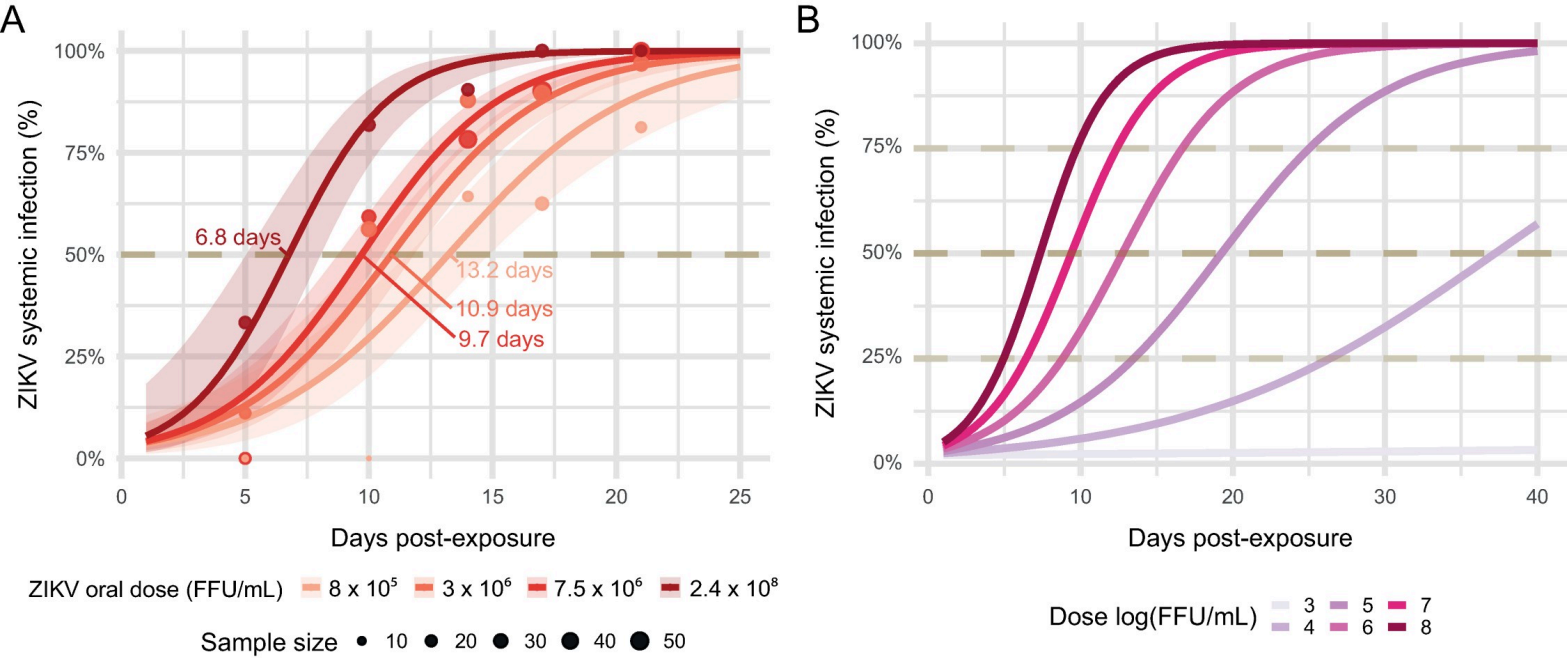
*Ae*. *albopictus* mosquito systemic infection rate dynamic post-virus exposure as a function of the oral dose. (A) Systemic infection rate dynamics observed experimentally. Dots correspond to systemic infection rates observed on *Ae*. *albopictus* mosquitoes at each infectious blood-meal titer. Dots size is proportional to the number of samples. The time-dependent effect of the virus dose on systemic infection rates was modelled using logistic regression. Lines correspond to fit values with their 95% confidence intervals displayed as ribbons. (B) Predicted systemic infection rate dynamics according to the virus dose and time post-virus exposure for a range of infectious blood meal titers (3 to 8 log10 FFU/mL).

Intra-host dynamics of systemic ZIKV infection was inferred for all virus doses by fitting a logistic model with the virus dose, the time post-exposure and their interaction as explanatory variables. Systemic infection prevalences were significantly influenced by the combination of the time post-exposure and the virus dose (analysis of deviance, interaction term, p-value = 0.03). Systemic infection prevalences saturated at 100% at 25 days post virus exposure for all virus doses but the lowest (8 × 10^5^ FFU/mL) for which 96% of mosquitoes had a systemic infection (Fig 4A). The estimated time required to reach 50% of systemic infections was 13.2 days (95% confidence interval: 11.5–15 days), 10.9 days (95% CI: 10–12 days), 9.7 days (95% CI: 9–10.5 days) and 6.8 days (95% CI: 5–8 days) for 8 × 10^5^ FFU/mL, 3 × 10^6^ FFU/mL, 7.5 × 10^6^ FFU/mL and 2.37 × 10^8^ FFU/mL infectious blood meal concentrations, respectively. Intra-mosquito systemic infection dynamics were inferred from a range of dose from 10^3^ to 10^8^ FFU/mL (Fig 4B). Median time estimates to reach 50% systemic infection were inversely correlated with the virus dose, ranging from 7.34 days to 37 days post virus exposure for the 10^8^ FFU/mL and 10^4^ FFU/mL doses, respectively. The lowest virus dose (10^3^ FFU/mL) was not sufficient to produce systemic infection in mosquito in the time range from which values were predicted (1–40 days).

### Relationship between intra-human ZIKV load dynamics and intra-mosquito systemic infection rate dynamics

We used our model of intra-human ZIKV load dynamics to generate ZIKV load dynamic variability by running the model on 1,000 independent iterations and sampling for each iteration a parameter value from a normal distribution in the form *N(μ =* mean parameter estimate, *σ2 = μ/6)*, with 6 being a scaling factor determined empirically based on the covering of patient data by the distribution of predicted viremia dynamics (black lines, Fig 1). The mean peak ZIKV viremia was 6.8 × 10^7^ ZIKV RNA copies per mL (5^th^ percentile: 3 × 10^7^ ZIKV RNA copies per mL, 95^th^ percentile: 1.3 × 10^8^ ZIKV RNA copies per mL) at a mean time of 2.5 days (5^th^ percentile: 2 days, 95^th^ percentile: 3 days) after infection, always reached before symptom onsets (black lines, Fig 1A). The median time of infectious ZIKV clearance post-infection was 9 days (25^th^ percentile: 8 days, 75^th^ percentile: 10 days, time to event analysis). At day 2 post-infection, a mean concentration of 7.3 × 10^4^ FFU/mL was observed (25^th^ percentile: 1.6 × 10^4^ FFU/mL, 75^th^ percentile: 1.2 × 10^5^ FFU/mL). The mean virus concentration dropped to 7 FFU/mL (25^th^ percentile: 0.1 FFU/mL, 75^th^ percentile: 2.5 FFU/mL) at day 9 post-infection (*i*.*e*., the median time of infectious ZIKV clearance post infection).

Mosquito infection probabilities were inferred throughout the dynamics of viremia in humans. Mosquito infection probability started at 0 and reached its peak at day 2 post-infection (22% according to model prediction using mean fit parameter values as determined in Fig 1A and 1B–the golden line in Fig 1C–with a mean 19.4% (25^th^ percentile: 9%, 75^th^ percentile: 29%) as calculated from the 1,000 iterations–black lines) to decrease rapidly and reach 0.3% at day 6 (i.e., time to symptom onset) with a mean of 0.7%, 25^th^ percentile: 0.2%, 75^th^ percentile: 0.86% as calculated from the 1,000 iterations.

Based on our model parameter estimation, the median time to reach systemic infection was the lowest at day 2 post-infection. At the peak of viremia, half of the infected mosquitoes would need 20.7 days (median 21 days, 25^th^ percentile: 19 days, 75^th^ percentile: 30 days, as calculated from the 1000 iterations) to develop a systemic infection in our experimental settings (Fig 1D). The median time to reach systemic infection was inferior to 50 days in only 20 out of the 1,000 iterations at the time of symptom onset in human (day 6 post-infection), and inferior to 30 days in only 3 out of the 1,000 iterations.

### Integration of the intra-host dynamics into outbreak simulations enhances realism to assess factors influencing outbreak initiation probability and scale

We implemented a stochastic agent-based model (ABM) to assess the epidemiological impact of within-host ZIKV dynamicsin dose-dependent manner using the R package nosoi [30]. Starting with one infected human in a population of susceptible hosts (humans and mosquitoes), the model simulates ZIKV transmissions by considering the dynamic nature of within-human virus loads and the associated probability of mosquito infection and virus transmission timeliness (*i*.*e*., Extrinsic Incubation Period, EIP). The model was run 100 independent times over 365 days over a range of mean individual mosquito biting rates: 1, 5, 10 and 60 independent mosquitoes biting per person per day.

**Fig 5:**
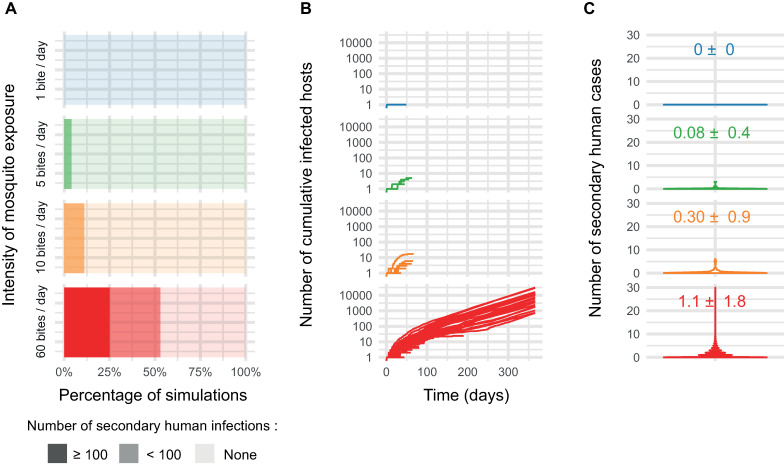
ZIKV outbreak simulations results according to levels of mosquito bites exposure and the consideration of the dose-dependent intra-mosquito ZIKV dynamic. Stochastic agent-based epidemiological simulations considering within-host infection dynamics on transmission probability during mosquito-human infectious contacts were performed in 100 independent replicates. A total of 4 mosquito bite intensity levels were tested: 1, 5, 10, and 60 bites per human per day. (A) Stacked proportions of outbreaks resulting in no secondary infected human host, infected human hosts < 100 and infected human hosts ≥ 100. (B) Cumulative number of infected humans over time. Each curve represents a simulation run. (C)- Violin plots showing the number of secondary cases values densities for each condition. Mean number of secondary cases ± Standard Deviation (SD) is represented in each panel.

The risk of ZIKV outbreak initiation varied significantly across mosquito biting rates densities (p-value = 0.0005, Fisher's Exact Test), with a probability of outbreak initiation success of 0%, 4%, 11% and 53% for 1, 5, 10, and 60 mosquito bites per person per day (Fig 5). Intensities of ZIKV outbreaks, as represented by the number of secondary human infections throughout of the epidemic, were also dependent on the intensity of mosquito bite exposure (Fig 5). Large-scale ZIKV outbreaks (*i*.*e*., outbreaks yielding more than 100 secondary human infections) occurred in 25% of the simulations at the highest bite intensity level only (60 bites per person per day). Outbreak dynamic was also related to the intensity of mosquito bite exposure. In our model, ZIKV outbreak terminated (i) in absence of infected humans in the synthetic population or (ii) when the maximum number of human or mosquito cases (i.e. 100,000 and 1,000,000 respectively) was reached. The maximum threshold of 1,000,000 mosquito infections was only reached at the highest mosquito biting intensity (*i*.*e*., 60 bites per person per day). The distribution of the number of secondary cases directly generated by one infectious case in a susceptible human population is a good indicator of an outbreak dynamic. Secondary cases values distributions across simulations were wide for all conditions, which reflect the stochastic nature of an outbreak. Mean secondary case values increased as a function of the mosquito biting intensity with a mean 0.0 ± 0.0 (Mean ± SD), 0.08 ± 0.4, 0.30 ± 0.9, and 1.1 ± 1.8 for 1, 5, 10, and 60 mosquito bites per person per day, respectively. The mean time separating a mosquito infection to the first virus transmission (EIP) was 24.2 ± 11.1 (Mean ± SD, calculated with 9 infectious mosquitoes over the 100 run replicates), 17.8 ± 9.4 (N = 35 infectious mosquitoes across replicates) and 17.9 ± 10.2 days (N = 238,862 mosquitoes across replicates) for the for 5, 10, and 60 mosquito bites per person per day, respectively (there was no infectious mosquitoes for the lowest bite density). These results are coherent with the median time distribution to reach systemic infection at day 2 post-infection calculated previously.

## Discussion

While the mosquito *Aedes hensilli* was strongly suspected of transmitting ZIKV during the Yap island outbreak [9], the yellow fever mosquito *Aedes aegypti* has been incriminated in all major outbreaks that have occurred in the Pacific Ocean, South-America or the Caribbean, especially in urban settings [31]. The Asian tiger mosquito *Ae*. *albopictus* is considered as the second most important vector of human viral pathogens. Originating from Asia, this species has spread throughout the world and is now present in almost all continents, including in tropical and temperate regions. Although considered as having more general blood-feeding preferences than *Ae*. *aegypti* (the latter has a strict anthropophilic behavior), *Ae*. *albopictus* is well adapted to the urban environment and can be an aggressive biter and an important nuisance for the human populations [32]. This species was found to be competent for ZIKV experimentally [23,26,31,33–36], and strongly suspected to sustain a ZIKV epidemic activity (African lineage) in Gabon in 2007 [8]. *Ae*. *albopictus* was also implicated recently in autochthonous ZIKV (Asian lineage) transmissions in the South of France [27], which provides clear confirmation of its vector potential. However, it is not known if *Ae*. *albopictus* could trigger large scale epidemics in areas where the species exists.

To explore this potential, we studied the dynamic nature of vector competence of *Ae*. *albopictus* for ZIKV. We assessed the link between ZIKV loads dynamic in humans and *Ae*. *albopictus* infection probability and systemic infection dynamics. One major originality of our study is to consider the inter-relationship between both within-hosts dynamics involved in the virus life-cycle. We aimed to provide a global and realistic view of *Ae*. *albopictus* vector competence for ZIKV by considering vector competence as time and virus dose-dependent. Our study revealed that the mosquito infection rate increases as a function of the infectious blood meal dose, independently of the time post-exposure. Infections rates were shown to be highly variable across studies: a meta-analysis of the literature that focused on *Ae*. albopictus*’* competence for ZIKV revealed an overall high estimated infection rate of 79% (95% CI 69%–89%) 7 days post-exposure with significant heterogeneity across studies [37]. To our knowledge, only two studies have assessed *Ae*. *albopictus* infection rates as a function of the ZIKV dose [25,26]. The oral median infection dose (ID50) estimated by these studies ranged from 6.1 to 6.8 log_10_ FFU/mL. Our ID50 estimate (5.6 log_10_ FFU/mL (95% CI: 5.3 log_10_ FFU/mL—5.8 log_10_ FFU/mL)) was in the same order of magnitude and suggested that the median infection dose in *Ae*. *albopictus* is not strongly influenced by mosquito populations and/or viral isolates. We revealed that these ID50 values are higher than the maximum ZIKV load estimates occurring in the course of the viremia post-infection in humans (which was estimated to be about 5 log_10_ FFU/mL). We estimated that the mosquito infection probability was around 20% at the peak of viremia in humans (mean concentration of 7.3 × 10^4^ FFU/mL at day 2 post-infection). This optimum probability of mosquito infection is very transient, and drops to 0.3% at day 6 post-infection in humans (*i*.*e*., average time to symptom onset).

Low systemic infection rates for ZIKV and *Ae*. *albopictus* were often revealed in several independent studies [23,24]. These results are in apparent contradiction with our findings showing levels of systemic infection rates that reach 100% in both Marseille (Metropolitan France) and La Réunion (Indian Ocean) populations from 5 log_10_ FFU/mL upwards. Similar high systemic infection rates were also described in the literature after 14 days post virus exposure and for ZIKV infectious doses above 5 log_10_ PFU/mL [25,26,36,38]. It is not straightforward to identify factors underlying these systemic infection rate discrepancies across studies. Mosquito vector competence for viruses was reported to differ according to mosquito populations [39,40], virus isolates [41], their combinations [42], or the virus dose [25,26]. More complex interactions between intrinsic (*e*.*g*., mosquito virome [43], endogenous non-retroviral elements [44,45], or bacterial microbiome [46]) or environmental (*e*.*g*., temperature [47,48], rearing or experimental settings) conditions might further impede comparisons across vector competence studies [49]. Systemic infection dynamic was strongly influenced by the infectious blood meal dose, with a 30 days shift of median time estimates to reach 50% systemic infection between 5 log_10_ FFU/mL and 8 log_10_ FFU/mL. At the peak of Zika viremia in humans, half of the infected mosquitoes would need around 21 days to develop a systemic infection in our experimental settings. Aside for highlighting the high risk of ZIKV transmission during the pre-sympotmatic stage of the infection, these results revealed a relatively low epidemic potential of *Ae*. *albopictus* for ZIKV. Tesla B. and colleagues have previously empirically demonstrated the dependency of infection rate, systemic infection and transmission dynamic on ZIKV oral viral dose using the *Ae*. *aegypti* mosquito model [28]. They estimated at 4.98 log_10_ Plaque Forming Units (PFU)/mL the infectious dose required to infect 50% of the mosquito population (ID50). By applying our analysis procedure (S1 File) on their raw data, we estimated the time estimates to reach 50% systemic infection to be 18.7, 14 and 8.2 days across their 3 replicates for a 5 log_10_ PFU/mL oral dose. By comparison, we estimated in *Ae*. *albopictus* the ID50 at 5.6 log_10_ FFU/mL and at 19.2 days the median systemic infection estimate for a similar ZIKV oral dose (5 log_10_ FFU/mL, different titration methods were applied between both studies). These results suggest that infection rates and intra-mosquito virus dynamics are similar across *Ae*. *aegypti* and *Ae*. *albopictus* mosquito species, despite the documented role of *Ae*. *aegypti* as the main vector during major ZIKV outbreaks. An overall low intrinsic ability of both species to transmit ZIKV in experimental settings has been previously reported [23], and other parameters might be at play to allow a sustained virus transmission on the field. It has been previously demonstrated that an urban, *Aedes aegypti*-borne, epidemic of yellow fever occurred in 1987 in Africa although the mosquito vector was relatively resistant to infection and transmitted the virus inefficiently. It was suggested that a high mosquito density can override a relatively low vector competence to sustain active virus transmissions [50].

Combined with blood feeding behaviours, mosquito bite rate and vector longevity, intra-mosquito dynamic is a powerful contributor to vectorial capacity (VC), a restatement of the basic reproductive rate (R_0_) of a vector-borne pathogen [51,52]. To approximate VC, we build a realistic vector-borne virus transmission framework throught the implementation of Agent-Based Simulations (ABM) based on the within-hosts dynamic inter-relationship. We revealed that the probability of ZIKV outbreak initiation, dynamics, and scale were strongly dependent on the intensity of mosquito bite exposure. The probability of a large ZIKV outbreak (≥ to 100 secondary human cases) to initiate ranged from 0% at 1 bite per person per day to 25% at 60 bite per person per day. The positive influence of the human biting rate parameter on VC has been well depicted by deterministic models representing VC, such as the Ross-Macdonald model [51,53,54]. In these models, vector competence is treated as a limiting factor (a proportion or probability) fixed in time and not influenced by other parameters. We here reached similar conclusions using a different and independent system that can easily integrate the stochastic and dynamic nature of outbreaks. It thus provides a more realistic scenario where vector competence is treated as a dynamic parameter influenced by the virus dose, as highlighted by our experimental results.

An almost unanimous consensus is prevailing on the existence of a low transmission efficiency (*i*.*e*., high transmission barrier) [22–24,26,34,35,55] in the *Ae*. *albopictus*/ZIKV couple. Variation of the transmission barrier permeability as a function of the virus dose or mosquito population was not addressed in our work. The presence of a transmission barrier would further scale down our predictions concerning the epidemic potential of *Ae*. *albopictus* for ZIKV but would not impact our main conclusions regarding the dependency of intra-mosquito ZIKV infection and systemic infection dynamic on the virus dose. Alternatively, many other factors that have not been considered here can enhance this potential. For instance, higher ZIKV load and prolonged duration of virus detection were encountered in capillary blood as compared to venous blood [56]. It has also been observed that infection rates in mosquitoes are higher when viremic hosts are used as the blood meal source, as compared to artificial infectious blood feeding [57]. Based on our prediction, any increase in virus dose intake by the mosquito would result in a dramatic rise of the epidemic potential of *Ae*. *albopictus* for ZIKV. In addition, transmission efficiency was reported to be strongly affected by temperature: median EIP was shown to shift from 9.6 days at 26°C to 5.1 days at 30°C for *Ae*. *aegypti* mosquitoes infected with ZIKV [47]. ZIKV might also have the opportunity to evolve towards shorter intra-mosquito dynamics, as had already occurred in other system [58]. Other ZIKV genotypes, such as those from the African lineage, might also lead to more permissive infection and systemic infection barriers in *Ae*. *albopictus* [59].

Our conclusions concerning the inter-relationship between within-hosts dynamics also rely on a strong hypothesis of similar Zika viremia dynamics profil in human and non-human primates. While clinical manifestations of arbovirus infections often differ from humans in non-human primates, both model appear to share similar estimates of duration of viremia [60,61]. Modelisation of Zika viremia in humans is challenging due to the paucity of data available. Viral loads can generally only be accessed in symptomatic patients after symptom onsets, and it is not straightforward to date the time of infection through an infectious mosquito bite with accuracy. A large proportion of ZIKV infections are asymptomatic, and virus loads dynamic remains elusive for this category of patients. In addition, we simulated a within-human viremia diversity by considering no-correlation across the several parameters of our model, which might not truly reflect the reality. We aimed to provide the best viremia model based on the few data that were available, and as such any deviations from our initial hypothesis might impact our predictions. However, any additional information provided by future studies would be easily integrated in our ABM model (provided in S2 File) to update our assessement of *Ae*. *albopictus* as a vector.

In summary, our results revealed a low epidemic potential of *Ae*. *albopictus* for ZIKV, independently of its tropical or temperate origin, which might explain the absence of large scale ZIKV epidemics in territories occupied by *Ae*. *albopictus*. This low epidemic potential however cannot be considered as an obstacle to autochthonous ZIKV transmissions as it can rise under favorable conditions (*e*.*g*., high mosquito density) to trigger large scale ZIKV outbreaks.

## Materials and methods

### Ethics statement

Institutional review board approval was granted by the Comité de Protection des Personnes Sud-Méditerranée I corresponding to the following study “Etude descriptive prospective de la maladie à virus Zika au sein de la communauté de défense des Forces Armées en Guyane” and was registered February 2016 under the number RCB: 2016-A00394-47. Written informed consent was obtained from each patient as required by the Comité de Protection des Personnes Sud-Méditerranée I.

### Zika virus viremia dynamic in human

#### Data for ZIKV plasma loads in human over time

Venous serum samples from 35 symptomatic Zika virus patients were collected at different time points post-symptoms onset. All patients were infected during a period ranging from March to September 2016 in French Guiana in the course of the ZIKV outbreak in the American continent starting in 2015. Twenty-one of these patients were included in a prospective study that described ZIKV genomic RNA load in sera samples collected sequentially from venous and skin capillary blood [56]. Thirteen additional patients from whom sera were only collected from venous blood were added in this study (virus loads for these patients can be considered here as primary data) [62]. ZIKV genomic RNA was extracted from 150 μL of each sample using the QIAamp Viral RNA kit (QIAGEN, Hilden, Germany). ZIKV loads, expressed as the number of genomic RNA copies per mL, were determined using the RealStar Zika Virus real-time reverse-transcription PCR (RT-PCR) Kit 1.0 (Altona Diagnostics GmbH, Hamburg, Germany) according to the manufacturer’s instructions and a standard curve generated using 10-fold serial dilutions of quantified total ZIKV RNA (28930015). All patients were followed up until the absence of ZIKV RNA detection in their venous serum.

Importantly, we had access to one sample collected one day before symptoms onset. To our knowledge, this represents the only human serum sample that has been collected in the pre-symptomatic stage of the disease so far. Only the quantification cycle (Cq) values could have been recovered for this patient because RT-PCR was performed without a standard curve. One ZIKV load (RNA copies/mL) value was also missing for another patient at his first time-point post-symptoms onset. Because these samples were no longer available at the time of data analyses, and because we consider that they were critical to model ZIKV viremia over time post infection, ZIKV loads (RNA copies/mL) were inferred from Cq values based on a linear regression analysis performed with RT-PCR data from the other samples and diluted standards. While performing absolute quantitation based on comparison of Cq values that were not obtained from the same experiment could lead to inaccurate estimates [63], we deliberately chose this approach because the same RT-PCR procedure was used across experiments independently of the presence of a standard curve. Patients without detectable virus load in their plasma were discarded from the analysis. A maximum of 4 plasma samples was available per patient. In order to improve parameters estimation in the subsequent model fitting step, a ZIKV plasma load dynamic was obtained post-symptom onsets by averaging ZIKV loads across 23 patients (S3 and S4 Files).

#### Fitting a virus load dynamic model to data

The within-human host plasma viral load dynamic was assessed using a target-cell limited model with a latent phase, as represented by a system of 4 ordinary differential equations described by Best *et al*. [29]:

$$\frac{dT}{dt}=-\beta VT;\;\frac{dI_{1}}{dt}=\beta VT-kI_{1};\;\frac{dI_{2}}{dt}=kI_{1}-\delta I_{2};\;\frac{dV}{dt}=pI_{2}-cV.$$

This model was used to model plasma Zika virus dynamics in nonhuman primates. *T*, *I*_*1*_, and *I*_*2*_ represent the concentration (cells per mL) of (i) uninfected target cells, (ii) infected target cells that do not produce virus yet, and (iii) productive infected cells, respectively. *V* represents the concentration of free virus particles (ZIKV RNA copies/mL). We assumed that infection is initiated by the introduction of *V*_*0*_ ZIKV RNA copies into the human host during the mosquito bite. Free viruses (*V*) infect target cells (*T*) at rate *β*TV. This constant infection rate of target cells per virion (*β*) is a limiting factor affecting the probability of a virion to enter a cell. Infected cells then enter in a latent phase (*I*_*1*_) where they do not produce virus and transition to a virus productive state (*I*_*2*_) at rate *k* per day, with 1/*k* being the average transition time from *I*_*1*_ to *I*_*2*_. Productively infected cells (*I*_*2*_) release free viruses at a rate *p* per day and die at a rate δ per day, where 1/δ is the average lifespan of productively infected cells. Free viruses are cleared at a rate *c* per day due to the effect of the immune response or virus decay. Target cell replenishment was ignored in the model because of the short-term nature of the infection.

The model was fitted to observed data using a Bayesian approach implemented in Stan [64] through the Rstan package [65] (S3 and S4 Files). A fixed incubation period of 6 days was added to each time post-exposure in our data prior to fit the model in order to standardize the time scale between our data and the model (*i*.*e*., our data are expressed in time post-symptom onset while the model start at the day of infection). This six-day incubation period corresponds to the rounded averaged median time of the ZIKV incubation period estimated from different independent studies (i.e. 5.9 days (95% credible interval, 4.4–7.6 days) for Lessler J. *et al*., 6.2 (95% CI 5.7––6.6) for Krow-Lucal E.R. *et al*., and 6.8 (95% CI, 5.8–7.7 days) for Fourié T. *et al*.) [66–68]. The lack of data before symptom onset precludes the estimation of a unique combination of model parameter values that could fit the data with a family of solutions. To address this issue, we used parameter estimates inferred from non-human primate viremia dynamics as strong priors in our model fitting process under the strong hypothesis that human and non-human viremia dynamics are closely related [69]. Prior distributions were adapted from Best *et al*. [29]. The initial virus load distribution was modified to be into the range of ZIKV titers found in *Aedes aegypti* saliva [70] (around 100 infectious viruses for an estimated volume of 10 nL of saliva [71]). The initial normal prior distributions for virus load and target cells concentration, denoted *V0* and *T0* respectively, were set to *V0*: *N(μ = 10*,*000*, *σ2 = 500)* and *T0*: *N(μ = 1e5*, *σ2 = 1e4)*, respectively. The initial concentration of infected cells, I_1_ and I_2_, was assumed to be zero. Parameters *β*, *k*, *δ*, *p* and *c* were sampled from the respective normal prior distributions *β*: *N(μ = 2e-7*, *σ2 = 1)*, *k*: *N(μ = 6*, *σ2 = 3)*, *δ*: *N(μ = 2*, *σ2 = 1)*, *p*: *N(μ = 25*,*000*, *σ2 = 2*,*500)* and *c*: *N(μ = 10*, *σ2 = 3)*. The model was run on four independent chains over 2,000 iterations with sampling beginning after 1,000 warmup iterations. Parameter estimates have an effective sample size > 1,000 and a potential scale reduction factor (*R-hat*) of 1, both of which ensure convergence and a lack of autocorrelation. Prior posterior overlaps (PPO) were calculated with the MCMCvis R package [72]. Parameters with a PPO >35% were not considered robustly identifiable [73].

Predicted ZIKV RNA loads (copies/mL) were converted into infectious virus loads (FFU/mL) by dividing the viremia predicted by the model by a fixed arbitrary value of 750. This value lies at the midrange of the RNA copies/PFU ratios (infectious titers were reported to be 500–1,000-fold less than ZIKV RNA copies concentrations) that were estimated in non-human primate plasma with a French Polynesian ZIKV isolate [74].

### Zika virus isolate

In this study, we used the ZIKV isolate SL1602 that was isolated from the plasma of a traveler returning from Suriname in 2016 [75]. This isolate belongs to the Asian lineage and is phylogenetically close to other viruses that have recently been isolated in the Americas [75]. The virus was passaged four times in C6/36 cells (*Aedes albopictus*) prior to its use for mosquito infections. To prepare virus stock, sub-confluent C6/36 cells were infected using a viral multiplicity of infection (MOI) of 0.1 (one infectious viral particle for 10 cells) in 25-cm^2^ culture flasks and incubated for 7 days at 28°C with 5 mL of Leibovitz’s L-15 medium supplemented with 0.1% penicillin (10,000 U/mL)/streptomycin (10,000 μg/mL) (Life Technologies, Grand Island, NY, USA), 1X non-essential amino acids (Life Technologies) and 2% fetal bovine serum (FBS, Life Technologies). At the end of the incubation, the cell culture medium was harvested, adjusted to 10% FBS, and pH ~8 with sodium bicarbonate, aliquoted, and stored at −80°C. An additional 5 mL of Leibovitz’s L-15 medium prepared as described above were added to infected confluent C6/36 cells and harvested 3 days later. This procedure increases virus titres in the stock solution.

Virus titration was performed by focus-forming assay (FFA) on one aliquot that has been stored at −80°C, as previously described with minor modifications [76]. This assay relies on inoculation of 10-fold dilutions of a sample onto a sub-confluent culture of C6/36 cells, followed by incubation and subsequent visualization of infectious foci by indirect immunofluorescence. Modifications to the previously published protocol include a 1-hour incubation step at 37°C with 40 μL/well of mouse anti-*Flavivirus* group antigen antibody clone D1-4G2-4-15 (Merck Millipore, Molsheim, France) diluted 1:200 in PBS + 1% bovine serum albumin (BSA) (Interchim, Montluçon, France). After another three washes in PBS, cells were incubated at 37°C for 30 min with 40 μL/well of a goat anti-mouse IgG (H+L), FITC-conjugated antibody (Merck Millipore). After three more washes in PBS and a final wash in ultrapure water, infectious foci were counted under a fluorescent microscope and converted into focus-forming units/mL (FFU/mL). The titre of the frozen virus stock was estimated at 6.35 × 10^5^ focus-forming units per mL (FFU/mL) for the first medium culture harvest and 2.75 × 10^8^ FFU/mL for the second medium culture harvest. All infectious experiments were conducted in a Biosafety Level-3 (BSL-3) insectary (IHU Méditerranée Infection, Marseille).

### Mosquito populations

**Fig 6:**
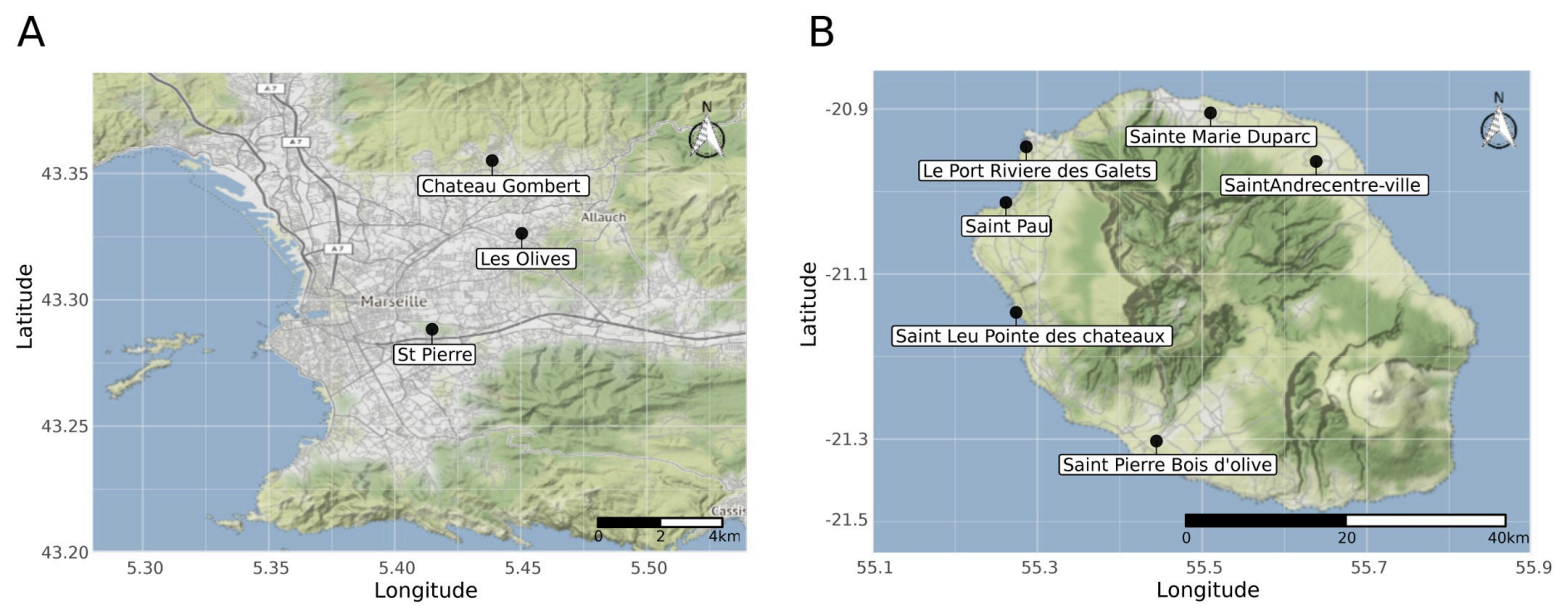
**Geographic location of the F0 mosquito collecting sites in Marseille (A) and Réunion island (B).** Mosquitoes from these sites were the wild progenitors of the Marseille (Metropolitan France) and La Réunion (Overseas France, Indian ocean) populations (4^th^ generation) used in this study. Source: Base map and data from OpenStreetMap and OpenStreetMap Foundation.

Two mosquito populations of *Aedes albopictus* species were used in this study, one from Marseille (Metropolitan France) and the other from La Réunion island, a French overseas territory in the Indian Ocean. *Ae*. *albopictus* mosquitoes from Marseille were collected both as eggs and adults using ovitraps and human landing catches in three different locations in Marseille city (5^th^, 12^th^, and 13^th^ districts) in June 2018. *Ae*. *albopictus* mosquitoes from La Réunion were all collected as eggs in June 2018 from six different localities named Sainte Marie Duparc, Saint André centre-ville, Saint Paul, Le Port Rivière des Galets, Saint Leu Pointe des châteaux, Saint Pierre Bois d’olive (Fig 6). A first-generation population was obtained from the eggs of adults originating from ovitraps or captured by human landing catches. For each population, larvae from different collection sites were combined, and adult mosquitoes were maintained in standardized insectary conditions (28°C, 75 ± 5% relative humidity, 16:8 hours light-dark cycle) until the 4^th^ generation by mass sib-mating and collective oviposition. Adult females from each population were blood-fed on human blood obtained from the Etablissement Français du Sang (EFS) through a membrane feeding system (Hemotek Ltd, Blackburn, UK) using pig intestine as the membrane. Access to human blood was based upon an agreement with the EFS. Eggs were hatched in reverse osmosis water, and larvae were reared with a standardized diet of fish food in 24 × 34 × 9 cm plastic trays at a density of about 400 larvae per tray. A maximum of 800 male and female adults was maintained in 24 × 24 × 24 cm screened cages with permanent access to a 10% sucrose solution.

### Experimental mosquito infections

Nine to 13-day old females were transferred to the BSL-3 and deprived of sucrose solution 24 hours before experimental virus exposure. Sixty to 80 females were confined into 80-mm high and 80/84 mm (inside/outside) diameter cardboard containers. Containers were sealed on the top with mosquito mesh and with a 65-mm high polystyrene piston covered up with a plasticized fabric that matches the inside diameter of the container at the bottom. Pistons of each container were rose before to infectious blood meal exposure to contain all mosquitoes in a tight space below the infectious blood meal. This procedure increases the yield of engorged mosquitoes. Females were allowed to feed for 20 min from an artificial feeding system (Hemotek) covered by a pig intestine membrane that contained the infectious blood meal maintained at 37°C. Feeders were placed on top of the mesh that sealed the containers. The infectious blood meal consisted of two volumes of washed human erythrocytes and one volume of viral suspension. Human erythrocytes were collected and washed one day before the experimental infection. An aliquot of the artificial blood meal was collected immediately before blood-feeding and titred by FFA, as described above but without a freezing step.

Experimental mosquito exposure to ZIKV was performed in three independent experiments. Mosquitoes from both Marseille and La Réunion populations were exposed to the same infectious blood meal in experiments 1 and 2. In experiment 3, mosquitoes from the La Réunion population only were exposed to two different infectious blood meal titers. Final ZIKV virus titres in blood meals were 7.5 × 10^6^ FFU/mL and 3 × 10^6^ FFU/mL for experiment 1 and 2, respectively and 2.37 × 10^8^ FFU/mL and 8 × 10^5^ FFU/mL for experiment 3. After virus exposure, fully engorged females were cold anesthetized and sorted on ice before being individually transferred to new cardboard containers and maintained under controlled conditions (28°C, 75 ± 5% relative humidity, 16:8 hours light-dark cycle with permanent access to a 10% sucrose solution).

### ZIKV RNA detection

ZIKV RNA was detected in mosquito bodies and heads after a fresh dissection from freeze-killed mosquitoes at 5,10, 14, 17, and 21 days post virus exposure (DPE) for both *Ae*. *albopictus* populations. The presence of ZIKV RNA in bodies indicates a midgut infection, while the presence of the virus RNA in mosquito heads indicates a systemic (disseminated) infection [41]. These two vector competence indices were determined qualitatively (i.e., presence or absence of virus in mosquito bodies and heads, respectively). Dissected mosquito heads and bodies were homogenized individually in 400 μL of lysis buffer (NucleoSpin 96 Virus Core Kit, Macherey-Nagel, Hoerdt, France) during three rounds of 20 sec at 5,000 rpm in a Precellys 24 mixer mill (Ozyme, Saint-Quentin-en-Yvelines, France). Viral RNA from individual organs was extracted using the NucleoSpin 96 Virus Core Kit (Macherey-Nagel) according to the manufacturer’s instructions. At the final step, viral RNA from each sample was eluted in 100 μL of RNase-free elution buffer.

Detection of ZIKV RNA was performed with an end-point reverse transcription polymerase chain reaction (RT-PCR) assay. ZIKV genomic RNA was first reverse transcribed to complementary DNA (cDNA) with random hexamers using M-MLV Reverse Transcriptase (Life Technologies) according to the manufacturer’s instructions. cDNA was amplified by 35 cycles of PCR using the set of primers targeting the RNA-dependent RNA polymerase NS5 gene: ZIKV-F: 5'-GCCATCTGGTATATGTGG-3' and ZIKV-R: 5'-CAAGACCAAAGGGGGAGCGGA-3'. Amplicons (393 bp) were visualized by electrophoresis on 1.5% agarose gels.

### Statistical analysis

The time-dependent effect of the mosquito population on mosquito body infection and systemic infection was analysed by logistic regression by considering each phenotype as a binary response variable. A full-factorial generalized linear model that included the time post-virus exposure and the mosquito population or the virus dose, depending on the analyses, was fitted to the data with a binomial error structure and a logit link function. Statistical significances of the predictors’ effects were assessed by comparing nested models for their changes in deviances (goodness of fit measure for a model) based on a chi-squared distribution. Mosquito populations could be compared for a single dose without the confounding effect of the experiment in experiments 1 and 2. The effect of the virus dose was however confounded with the experiment effect when analysing the virus dose effect on intra-mosquito infection and systemic infection dynamics.

Probabilities of infection or systemic infection according to a virus dose or/and time post virus exposure were estimated with a logistic model in the mathematical form: $\log\left(\frac{P}{1-P}\right)=\beta_{0}+\beta_{1}\times X1+\beta_{2}\times X2+\beta_{3}\times X1\times X2+\varepsilon$, where P denotes the phenotype probability, *β*_0_ the Y-intercept when all model coefficients equal 0, ε the error term, and *X*_*i*_ and *β*_*i*_ the explanatory variables with their associated coefficients. Infection probabilities and median systemic infection doses were calculated based on logistic regression parameter estimates (S4 and S5 Files). Time to event analysis was performed using the packages survival [77] and survminer [78]. Fisher's Exact Test with simulated p-value (based on 2000 replicates) was performed to assess the significance of ZIKV outbreak initiation success across biting rates densities. All statistical analyses were performed in the statistical environment R [79]. Figures were made using the package ggplot2 [80] and the Tidyverse environment [81].

### Epidemiological modeling

#### Model overview

To explore how variation in human viremia dynamics can impact the epidemic potential of ZIKV, we performed a series of stochastic agent-based epidemiological simulations using the R package nosoi [82], similar to the simulations performed in Fontaine et al. [41], as a specific branch available on nosoi’s GitHub page (https://github.com/slequime/nosoi/tree/fontaine) (S2 File). This framework allows us to take into account within-host infection dynamics on transmission probability during mosquito-to-human and human-to-mosquito infectious contacts and place it in its full epidemiological context. We assumed that transmission could only happen between either an infected human and an uninfected mosquito or between an infected mosquito and an uninfected human. We thus ignored both sexual and vertical transmission as their epidemiological importance is unclear in both mosquitoes and humans, as well as excluded potential superinfections. We also assumed no particular structure in the host or mosquito population. It was assumed that human agents do not die from the infection and leave the simulation when they cure their infection after 12 days. Mosquitoes had a daily probability to die empirically set at 0.15 as used previously [41,83].

### Human-to-mosquito transmissions

Each human agent was exposed to a number of biting load drawn from a Poisson distribution with a mean value empirically set to 1, 5, 10 or 60 individual mosquito bites per person per day. Sixty bites per person per day is relevant to outdoor *Ae*. *albopictus* females biting activity on La Réunion island [84].

The target-cell limited model with a latent phase was used to simulate Zika viremia in our synthetic environment. For each individual, parameter values were drawn from normal distributions or truncated normal distributions (to avoid negative values) centered on mean parameter estimates (*μ*, refer to the “*Zika virus viremia dynamic in human*” section of the Materials and Methods) with standard deviations (*σ*) equal to mean parameter estimates divided by 6, with 6 being a scaling factor determined empirically based on the covering of patient data by the distribution of predicted viremia dynamics. ZIKV RNA loads (copies/mL) were converted into infectious virus loads (FFU/mL) by dividing by a value drawn from a uniform distribution ranging from 500 to 1,000, based on results showing that infectious titres were 500–1,000-fold less than ZIKV RNA copies concentrations in non-human primate plasma [74]. Mosquito infection probability was inferred for each mosquito-infected human contacts based on the simulated infectious virus dose at the time post-human infection. The probability of human-to-mosquito transmission was inferred from the experimental exposure of *Ae*. *albopictus* to ZIKV using our logistic model predictions.

### Mosquito-to-human transmissions

A gonotrophic cycle duration, separating two blood-meals, was drawn for each mosquito in a Poisson distribution with a mean of 4 days [85]. Mosquitoes were only allowed to bite one new human agent at times corresponding to multiples of their gonotrophic cycles durations.

Mosquito transmission probability were inferred for each contact from our full-factorial logistic model with the simulated infectious virus dose that initiated the infection in the mosquito and time post-infection as parameters. Time to systemic infection was used as a proxy for time to transmission (*i*.*e*., the extrinsic incubation period, referred to as EIP). We assumed a lack of salivary gland escape barrier and that the virus presence in mosquito head tissues indicated the potential for onward transmission. For each mosquito, an individual EIP value acts as the threshold for active transmission (when time post-infection is greater or equal to this EIP value, then the mosquito can transmit the infection). This individual EIP value was inferred from our time- and dose-dependent logistic regression based on this equation:

$$DD50=(\log\left(\frac{-P}{P-1.001}\right)-\beta_{0}-\beta_{1}\times X1)/(\beta_{2}+\beta_{3}\times X1)$$

where P = 0.5 (i.e., the median systemic dissemination probability), *β*_0_ is the Y-intercept value (4.17483), *β*_1_ (0.10116), *β*_2_ (-0.25564) and *β*_3_ (0.08924) are model coefficients associated to the virus dose, time post virus exposure and their interaction, respectively. X1 represents the virus dose value.

#### Implementation

These simulations were run in R version 3.6.4 using the packages foreach [86], doParallel [87], and nosoi [82]; complete R script used is available as S5 File. Briefly, simulations started with one infected human and were run for 365 days or until the allowed number of infected individuals was reached (100,000 humans or 1,000,000 mosquitoes respectively). Each condition was run in 100 independent replicate simulations.

## Supporting information

S1 Fig Markov chain Monte Carlo (MCMC) diagnostic plots for the ZIKV load dynamic model fitting. Trace plots of Markov chain Monte Carlo (MCMC) output for each of the 7 model parameters, with T0: the initial uninfected target cells concentration (cells per mL), V0: initial concentration of free virus particles (ZIKV RNA copies/mL), *β*: the infection rate constant of target cells per virion (per mL per day), *k*: rate of cell transition from a non-productive sate to a virus productive state (per day), *δ*: dying rate of productively infected cells (per day), *p*: free virus releasing rate by productively infected cells (per day) and *c*: virus clearance rate (per day). The model was run on 4 independent chains. The Rhat statistic, the number of effective samples, and the prior posterior overlap (PPO in %) are represented for each parameter.
(TIF)

S2 Fig Body infection prevalence of *Ae*. *albopictus* mosquitoes, according to ZIKV titers in the infectious blood meal. Percentages of body infections over time post-ZIKV exposure are represented at 4 infectious blood meal titer: 8 × 10^5^ FFU/mL, 3 × 10^6^ FFU/mL, 7.5 × 10^6^ FFU/mL and 2.4 × 10^8^ FFU/mL. 95% confidence intervals are indicated with the error bars, and prevalences averaged across time points are represented with a dashed line for each virus dose.
(TIF)

S1 File R code related to the intra-mosquito ZIKV dynamic analysis. The file provides code lines used to analyze the experimental data and perform data visualization and takes S5 File as an input file. This is a an R Markdown file created using RStudio, an open source Integrated Development Environment (IDE) for the R programming language. It contains YAML metadata, markdown-formatted plain text, and chunks of R code that can be rendered using RStudio.
(RMD)

S2 File R code used to perform the in silico epidemiological simulations with the nosoi R package. (R)

S3 File Database with patient identification number, the declared dates of symptoms, and blood sampling from the 35 patients infected with ZIKV included in our study. Quantification cycle (Cq) values for each sample and the corresponding ZIKV RNA concentration in copy/mL is provided when available.
(TXT)

S4 File R code related to the intra-human ZIKV dynamic analysis. The file provides code lines used to analyze the experimental data and perform data visualization. This is a an R Markdown file created using RStudio, an open source Integrated Development Environment (IDE) for the R programming language. It contains YAML metadata, markdown-formatted plain text, and chunks of R code that can be rendered using RStudio.
(RMD)

S5 File Raw vector competence data. Each line represents an individual mosquito and the columns are the experimental conditions, and phenotypes. From left to right, the columns indicate the mosquito identification number, the mosquito population (M = Marseille, LR = La Réunion island), number of days post-exposure to the infectious blood meal (dpe), the infectious blood meal concentration (FFU/mL), the body infection status, the head infection status (1 = presence of virus), and the experiment.
(TXT)

## References

[1] Kraemer MUG, Reiner RC, Brady OJ, Messina JP, Gilbert M, Pigott DM, et al Past and future spread of the arbovirus vectors *Aedes aegypti* and *Aedes albopictus*. Nat Microbiol. 2019;4: 854–863. 10.1038/s41564-019-0376-y 30833735 PMC6522366

[2] Gratz NG. Critical review of the vector status of *Aedes albopictus*. Med Vet Entomol. 2004;18: 215–227. 10.1111/j.0269-283X.2004.00513.x 15347388

[3] Armbruster PA. Photoperiodic Diapause and the Establishment of *Aedes albopictus* (Diptera: Culicidae) in North America. J Med Entomol. 2016;53: 1013–1023. 10.1093/jme/tjw037 27354438 PMC5013814

[4] Denlinger DL, Armbruster PA. Mosquito diapause. Annu Rev Entomol. 2014;59: 73–93. 10.1146/annurev-ento-011613-162023 24160427

[5] Juliano SA, Lounibos LP, O’Meara GF. A field test for competitive effects of *Aedes albopictus* on *A*. *aegypti* in South Florida: differences between sites of coexistence and exclusion? Oecologia. 2004;139: 583–593. 10.1007/s00442-004-1532-4 15024640 PMC1906877

[6] Hobbs JH, Hughes EA, Eichold BH. Replacement of *Aedes aegypti* by *Aedes albopictus* in Mobile, Alabama. J Am Mosq Control Assoc. 1991;7: 488–489. 1791461

[7] O’Meara GF, Evans LF, Gettman AD, Cuda JP. Spread of *Aedes albopictus* and decline of *Ae*. *aegypti* (Diptera: Culicidae) in Florida. J Med Entomol. 1995;32: 554–562. 10.1093/jmedent/32.4.554 7650719

[8] Grard G, Caron M, Mombo IM, Nkoghe D, Mboui Ondo S, Jiolle D, et al Zika virus in Gabon (Central Africa)—2007: a new threat from *Aedes albopictus*? PLoS Negl Trop Dis. 2014;8: e2681 10.1371/journal.pntd.0002681 24516683 PMC3916288

[9] Duffy MR, Chen T-H, Hancock WT, Powers AM, Kool JL, Lanciotti RS, et al Zika virus outbreak on Yap Island, Federated States of Micronesia. N Engl J Med. 2009;360: 2536–2543. 10.1056/NEJMoa0805715 19516034

[10] Musso D, Nilles EJ, Cao-Lormeau V-M. Rapid spread of emerging Zika virus in the Pacific area. Clin Microbiol Infect. 2014;20: O595–596. 10.1111/1469-0691.12707 24909208

[11] Jouannic J-M, Friszer S, Leparc-Goffart I, Garel C, Eyrolle-Guignot D. Zika virus infection in French Polynesia. Lancet. 2016;387: 1051–1052. 10.1016/S0140-6736(16)00625-5 26944027

[12] Lowe R, Barcellos C, Brasil P, Cruz OG, Honório NA, Kuper H, et al The Zika Virus Epidemic in Brazil: From Discovery to Future Implications. Int J Environ Res Public Health. 2018;15 10.3390/ijerph15010096 29315224 PMC5800195

[13] Silva GS, Richards GA, Baker T, Hidalgo J, Jiménez JIS, Amin P, et al Zika virus: Report from the task force on tropical diseases by the world Federation of Societies of intensive and critical care medicine. J Crit Care. 2018;46: 106–109. 10.1016/j.jcrc.2018.03.030 29779827

[14] Rawal G, Yadav S, Kumar R. Zika virus: An overview. J Family Med Prim Care. 2016;5: 523–527. 10.4103/2249-4863.197256 28217576 PMC5290753

[15] Renault P, Solet J-L, Sissoko D, Balleydier E, Larrieu S, Filleul L, et al A major epidemic of chikungunya virus infection on Reunion Island, France, 2005–2006. Am J Trop Med Hyg. 2007;77: 727–731. 17978079

[16] Pascalis H, Turpin J, Roche M, Krejbich P, Gadea G, Nten CA, et al The epidemic of Dengue virus type-2 Cosmopolitan genotype on Reunion Island relates to its active circulation in the Southwestern Indian Ocean neighboring islands. Heliyon. 2019;5: e01455 10.1016/j.heliyon.2019.e01455 31008393 PMC6458493

[17] Vasquez V, Haddad E, Perignon A, Jaureguiberry S, Brichler S, Leparc-Goffart I, et al Dengue, chikungunya, and Zika virus infections imported to Paris between 2009 and 2016: Characteristics and correlation with outbreaks in the French overseas territories of Guadeloupe and Martinique. Int J Infect Dis. 2018;72: 34–39. 10.1016/j.ijid.2018.05.007 29782922

[18] Succo T, Leparc-Goffart I, Ferré J-B, Roiz D, Broche B, Maquart M, et al Autochthonous dengue outbreak in Nîmes, South of France, July to September 2015. Euro Surveill. 2016;21 10.2807/1560-7917.ES.2016.21.21.30240 27254729

[19] Tomasello D, Schlagenhauf P. Chikungunya and dengue autochthonous cases in Europe, 2007–2012. Travel Med Infect Dis. 2013;11: 274–284. 10.1016/j.tmaid.2013.07.006 23962447

[20] Rezza G, Nicoletti L, Angelini R, Romi R, Finarelli AC, Panning M, et al Infection with chikungunya virus in Italy: an outbreak in a temperate region. Lancet. 2007;370: 1840–1846. 10.1016/S0140-6736(07)61779-6 18061059

[21] Vega-Rua A, Zouache K, Caro V, Diancourt L, Delaunay P, Grandadam M, et al High efficiency of temperate *Aedes albopictus* to transmit chikungunya and dengue viruses in the Southeast of France. PLoS ONE. 2013;8: e59716 10.1371/journal.pone.0059716 23527259 PMC3601061

[22] González MA, Pavan MG, Fernandes RS, Busquets N, David MR, Lourenço-Oliveira R, et al Limited risk of Zika virus transmission by five *Aedes albopictus* populations from Spain. Parasit Vectors. 2019;12: 150 10.1186/s13071-019-3359-1 30922370 PMC6440144

[23] Chouin-Carneiro T, Vega-Rua A, Vazeille M, Yebakima A, Girod R, Goindin D, et al Differential Susceptibilities of *Aedes aegypti* and *Aedes albopictus* from the Americas to Zika Virus. PLoS Negl Trop Dis. 2016;10: e0004543 10.1371/journal.pntd.0004543 26938868 PMC4777396

[24] Vazeille M, Dehecq J-S, Failloux A-B. Vectorial status of the Asian tiger mosquito *Aedes albopictus* of La Réunion Island for Zika virus. Med Vet Entomol. 2018;32: 251–254. 10.1111/mve.12284 29194706

[25] Azar SR, Roundy CM, Rossi SL, Huang JH, Leal G, Yun R, et al Differential Vector Competency of *Aedes albopictus* Populations from the Americas for Zika Virus. Am J Trop Med Hyg. 2017;97: 330–339. 10.4269/ajtmh.16-0969 28829735 PMC5544086

[26] Ciota AT, Bialosuknia SM, Zink SD, Brecher M, Ehrbar DJ, Morrissette MN, et al Effects of Zika Virus Strain and *Aedes* Mosquito Species on Vector Competence. Emerging Infect Dis. 2017;23: 1110–1117. 10.3201/eid2307.161633 28430564 PMC5512477

[27] Durand GA, Piorkowski G, Thirion L, Ninove L, Giron S, Zandotti C, et al Vector-Borne Transmission of the Zika Virus Asian Genotype in Europe. Viruses. 2020;12 10.3390/v12030296 32182748 PMC7150815

[28] Tesla B, Demakovsky LR, Packiam HS, Mordecai EA, Rodríguez AD, Bonds MH, et al Estimating the effects of variation in viremia on mosquito susceptibility, infectiousness, and R0 of Zika in *Aedes aegypti*. PLoS Negl Trop Dis. 2018;12: e0006733 10.1371/journal.pntd.0006733 30133450 PMC6122838

[29] Best K, Guedj J, Madelain V, de Lamballerie X, Lim S-Y, Osuna CE, et al Zika plasma viral dynamics in nonhuman primates provides insights into early infection and antiviral strategies. Proc Natl Acad Sci USA. 2017;114: 8847–8852. 10.1073/pnas.1704011114 28765371 PMC5565429

[30] Lequime S, Bastide P, Dellicour S, Lemey P, Baele G. nosoi: A stochastic agent-based transmission chain simulation framework in r. Johnson L, editor. Methods Ecol Evol. 2020;11: 1002–1007. 10.1111/2041-210X.13422 32983401 PMC7496779

[31] Epelboin Y, Talaga S, Epelboin L, Dusfour I. Zika virus: An updated review of competent or naturally infected mosquitoes. PLoS Negl Trop Dis. 2017;11: e0005933 10.1371/journal.pntd.0005933 29145400 PMC5690600

[32] Kamgang B, Nchoutpouen E, Simard F, Paupy C. Notes on the blood-feeding behavior of Aedes albopictus (Diptera: Culicidae) in Cameroon. Parasit Vectors. 2012;5: 57 10.1186/1756-3305-5-57 22433236 PMC3342132

[33] Dick GWA, Kitchen SF, Haddow AJ. Zika virus. I. Isolations and serological specificity. Trans R Soc Trop Med Hyg. 1952;46: 509–520. 10.1016/0035-9203(52)90042-4 12995440

[34] Di Luca M, Severini F, Toma L, Boccolini D, Romi R, Remoli ME, et al Experimental studies of susceptibility of Italian *Aedes albopictus* to Zika virus. Euro Surveill. 2016;21 10.2807/1560-7917.ES.2016.21.18.30223 27171034

[35] Heitmann A, Jansen S, Lühken R, Leggewie M, Badusche M, Pluskota B, et al Experimental transmission of Zika virus by mosquitoes from central Europe. Euro Surveill. 2017;22 10.2807/1560-7917.ES.2017.22.2.30437 28106528 PMC5404485

[36] Gutiérrez-López R, Bialosuknia SM, Ciota AT, Montalvo T, Martínez-de la Puente J, Gangoso L, et al Vector Competence of *Aedes caspius* and *Ae*. *albopictus* Mosquitoes for Zika Virus, Spain. Emerging Infect Dis. 2019;25: 346–348. 10.3201/eid2502.171123 30666939 PMC6346449

[37] McKenzie BA, Wilson AE, Zohdy S. *Aedes albopictus* is a competent vector of Zika virus: A meta-analysis. PLoS ONE. 2019;14: e0216794 10.1371/journal.pone.0216794 31112569 PMC6528984

[38] Wong P-SJ, Li MI, Chong C-S, Ng L-C, Tan C-H. *Aedes* (Stegomyia) *albopictus* (Skuse): a potential vector of Zika virus in Singapore. PLoS Negl Trop Dis. 2013;7: e2348 10.1371/journal.pntd.0002348 23936579 PMC3731215

[39] Bennett KE, Olson KE, Muñoz M de L, Fernandez-Salas I, Farfan-Ale JA, Higgs S, et al Variation in vector competence for dengue 2 virus among 24 collections of *Aedes aegypti* from Mexico and the United States. Am J Trop Med Hyg. 2002;67: 85–92. 10.4269/ajtmh.2002.67.85 12363070

[40] Gubler DJ, Nalim S, Tan R, Saipan H, Sulianti Saroso J. Variation in susceptibility to oral infection with dengue viruses among geographic strains of *Aedes aegypti*. Am J Trop Med Hyg. 1979;28: 1045–1052. 10.4269/ajtmh.1979.28.1045 507282

[41] Fontaine A, Lequime S, Moltini-Conclois I, Jiolle D, Leparc-Goffart I, Reiner RC, et al Epidemiological significance of dengue virus genetic variation in mosquito infection dynamics. PLoS Pathog. 2018;14: e1007187 10.1371/journal.ppat.1007187 30005085 PMC6059494

[42] Fansiri T, Fontaine A, Diancourt L, Caro V, Thaisomboonsuk B, Richardson JH, et al Genetic mapping of specific interactions between *Aedes aegypti* mosquitoes and dengue viruses. PLoS Genet. 2013;9: e1003621 10.1371/journal.pgen.1003621 23935524 PMC3731226

[43] Baidaliuk A, Miot EF, Lequime S, Moltini-Conclois I, Delaigue F, Dabo S, et al Cell-Fusing Agent Virus Reduces Arbovirus Dissemination in *Aedes aegypti* Mosquitoes In Vivo. J Virol. 2019;93 10.1128/JVI.00705-19 31243123 PMC6714787

[44] Houé V, Bonizzoni M, Failloux A-B. Endogenous non-retroviral elements in genomes of *Aedes* mosquitoes and vector competence. Emerg Microbes Infect. 2019;8: 542–555. 10.1080/22221751.2019.1599302 30938223 PMC6455143

[45] Suzuki Y, Baidaliuk A, Miesen P, Frangeul L, Crist AB, Merkling SH, et al Non-retroviral endogenous viral element limits cognate virus replication in *Aedes aegypti* ovaries. Microbiology; 2020 3 10.1016/j.cub.2020.06.057 32679098 PMC7522710

[46] Monteiro VVS, Navegantes-Lima KC, de Lemos AB, da Silva GL, de Souza Gomes R, Reis JF, et al *Aedes*-Chikungunya Virus Interaction: Key Role of Vector Midguts Microbiota and Its Saliva in the Host Infection. Front Microbiol. 2019;10: 492 10.3389/fmicb.2019.00492 31024463 PMC6467098

[47] Winokur OC, Main BJ, Nicholson J, Barker CM. Impact of temperature on the extrinsic incubation period of Zika virus in *Aedes aegypti*. PLoS Negl Trop Dis. 2020;14: e0008047 10.1371/journal.pntd.0008047 32187187 PMC7105136

[48] Carrington LB, Armijos MV, Lambrechts L, Scott TW. Fluctuations at a low mean temperature accelerate dengue virus transmission by *Aedes aegypti*. PLoS Negl Trop Dis. 2013;7: e2190 10.1371/journal.pntd.0002190 23638208 PMC3636080

[49] Azar SR, Weaver SC. Vector Competence: What Has Zika Virus Taught Us? Viruses. 2019;11 10.3390/v11090867 31533267 PMC6784050

[50] Miller BR, Monath TP, Tabachnick WJ, Ezike VI. Epidemic yellow fever caused by an incompetent mosquito vector. Trop Med Parasitol. 1989;40: 396–399. 2623418

[51] Macdonald G. Epidemiologic models in studies of vectorborne diseases. Public Health Rep. 1961;76: 753–764. 13764730 PMC1929763

[52] Kramer LD, Ciota AT. Dissecting vectorial capacity for mosquito-borne viruses. Curr Opin Virol. 2015;15: 112–118. 10.1016/j.coviro.2015.10.003 26569343 PMC4688158

[53] Brady OJ, Godfray HCJ, Tatem AJ, Gething PW, Cohen JM, McKenzie FE, et al Vectorial capacity and vector control: reconsidering sensitivity to parameters for malaria elimination. Trans R Soc Trop Med Hyg. 2016;110: 107–117. 10.1093/trstmh/trv113 26822603 PMC4731004

[54] Smith DL, Battle KE, Hay SI, Barker CM, Scott TW, McKenzie FE. Ross, macdonald, and a theory for the dynamics and control of mosquito-transmitted pathogens. PLoS Pathog. 2012;8: e1002588 10.1371/journal.ppat.1002588 22496640 PMC3320609

[55] Ryckebusch F, Berthet M, Missé D, Choumet V. Infection of a French Population of *Aedes albopictus* and of *Aedes aegypti* (Paea Strain) with Zika Virus Reveals Low Transmission Rates to These Vectors’ Saliva. Int J Mol Sci. 2017;18 10.3390/ijms18112384 29125545 PMC5713353

[56] Matheus S, de Laval F, Moua D, N’Guyen C, Martinez E, Rousset D, et al Zika Virus Persistence and Higher Viral Loads in Cutaneous Capillaries Than in Venous Blood. Emerging Infect Dis. 2017;23 10.3201/eid2311.170337 28930015 PMC5652410

[57] Duong V, Lambrechts L, Paul RE, Ly S, Lay RS, Long KC, et al Asymptomatic humans transmit dengue virus to mosquitoes. Proc Natl Acad Sci USA. 2015;112: 14688–14693. 10.1073/pnas.1508114112 26553981 PMC4664300

[58] Tsetsarkin KA, Vanlandingham DL, McGee CE, Higgs S. A single mutation in chikungunya virus affects vector specificity and epidemic potential. PLoS Pathog. 2007;3: e201 10.1371/journal.ppat.0030201 18069894 PMC2134949

[59] Vazeille M, Madec Y, Mousson L, Bellone R, Barré-Cardi H, Sousa CA, et al Zika virus threshold determines transmission by European *Aedes albopictus* mosquitoes. Emerg Microbes Infect. 2019;8: 1668–1678. 10.1080/22221751.2019.1689797 31735122 PMC6882490

[60] Clark KB, Onlamoon N, Hsiao H-M, Perng GC, Villinger F. Can non-human primates serve as models for investigating dengue disease pathogenesis? Front Microbiol. 2013;4 10.3389/fmicb.2013.00004 24130557 PMC3795305

[61] Althouse BM, Durbin AP, Hanley KA, Halstead SB, Weaver SC, Cummings DAT. Viral kinetics of primary dengue virus infection in non-human primates: A systematic review and individual pooled analysis. Virology. 2014;452–453: 237–246. 10.1016/j.virol.2014.01.015 24606701 PMC4578724

[62] de Laval F, d’Aubigny H, Mathéus S, Labrousse T, Ensargueix AL, Lorenzi EM, et al Evolution of symptoms and quality of life during Zika virus infection: A 1-year prospective cohort study. J Clin Virol. 2018;109: 57–62. 10.1016/j.jcv.2018.09.015 30523784

[63] Taylor SC, Nadeau K, Abbasi M, Lachance C, Nguyen M, Fenrich J. The Ultimate qPCR Experiment: Producing Publication Quality, Reproducible Data the First Time. Trends Biotechnol. 2019;37: 761–774. 10.1016/j.tibtech.2018.12.002 30654913

[64] Carpenter B, Gelman A, Hoffman MD, Lee D, Goodrich B, Betancourt M, et al *Stan*: A Probabilistic Programming Language. J Stat Soft. 2017;76 10.18637/jss.v076.i01 PMC9788645 36568334

[65] Stan Development Team. RStan: the R interface to Stan. R package http://mc-stan.org. 2018. Available: http://mc-stan.org

[66] Fourié T, Grard G, Leparc-Goffart I, Briolant S, Fontaine A. Variability of Zika Virus Incubation Period in Humans. Open Forum Infect Dis. 2018;5: ofy261 10.1093/ofid/ofy261 30397624 PMC6207619

[67] Krow-Lucal ER, Biggerstaff BJ, Staples JE. Estimated Incubation Period for Zika Virus Disease. Emerging Infect Dis. 2017;23: 841–845. 10.3201/eid2305.161715 28277198 PMC5403043

[68] Lessler J, Ott CT, Carcelen AC, Konikoff JM, Williamson J, Bi Q, et al Times to key events in Zika virus infection and implications for blood donation: a systematic review. Bull World Health Organ. 2016;94: 841–849. 10.2471/BLT.16.174540 27821887 PMC5096355

[69] Estes JD, Wong SW, Brenchley JM. Nonhuman primate models of human viral infections. Nat Rev Immunol. 2018;18: 390–404. 10.1038/s41577-018-0005-7 29556017 PMC5970954

[70] Roundy CM, Azar SR, Rossi SL, Huang JH, Leal G, Yun R, et al Variation in *Aedes aegypti* Mosquito Competence for Zika Virus Transmission. Emerging Infect Dis. 2017;23: 625–632. 10.3201/eid2304.161484 28287375 PMC5367433

[71] Sanchez-Vargas I, Harrington LC, Black WC, Olson KE. Analysis of Salivary Glands and Saliva from *Aedes albopictus* and *Aedes aegypti* Infected with Chikungunya Viruses. Insects. 2019;10 10.3390/insects10020039 30717086 PMC6410068

[72] Youngflesh C. MCMCvis: Tools to Visualize, Manipulate, and Summarize MCMC Output. JOSS. 2018;3: 640 10.21105/joss.00640

[73] Gimenez O, Morgan BJT, Brooks SP. Weak Identifiability in Models for Mark-Recapture-Recovery Data Springer Springer; Available: https://link.springer.com/chapter/10.1007/978-0-387-78151-8_48

[74] Dudley DM, Aliota MT, Mohr EL, Weiler AM, Lehrer-Brey G, Weisgrau KL, et al A rhesus macaque model of Asian-lineage Zika virus infection. Nat Commun. 2016;7: 12204 10.1038/ncomms12204 27352279 PMC4931337

[75] van Boheemen S, Tas A, Anvar SY, van Grootveld R, Albulescu IC, Bauer MP, et al Quasispecies composition and evolution of a typical Zika virus clinical isolate from Suriname. Sci Rep. 2017;7: 2368 10.1038/s41598-017-02652-w 28539654 PMC5443807

[76] Fontaine A, Jiolle D, Moltini-Conclois I, Lequime S, Lambrechts L. Excretion of dengue virus RNA by *Aedes aegypti* allows non-destructive monitoring of viral dissemination in individual mosquitoes. Sci Rep. 2016;6: 24885 10.1038/srep24885 27117953 PMC4846815

[77] Therneau T M. A Package for Survival Analysis in R. 2020. Available: https://CRAN.R-project.org/package=survival>

[78] Kassambara A, Kosinski M, Biecek P. survminer: Drawing Survival Curves using “ggplot2.” 2019 Available: https://CRAN.R-project.org/package=survminer

[79] R Core Team. R: A Language and Environment for Statistical Computing R Foundation for Statistical Computing, Vienna, Austria; 2019 Available: https://www.R-project.org/

[80] Wickham H. ggplot2: elegant graphics for data analysis Second edition Cham: Springer; 2016.

[81] Wickham H, Averick M, Bryan J, Chang W, McGowan L, François R, et al Welcome to the Tidyverse. JOSS. 2019;4: 1686 10.21105/joss.01686

[82] Lequime S, Bastide P, Dellicour S, Lemey P, Baele G. nosoi: a stochastic agent-based transmission chain simulation framework in R. Ecology; 2020 3 10.1111/2041-210X.13422 32983401 PMC7496779

[83] Favier C, Schmit D, Müller-Graf CDM, Cazelles B, Degallier N, Mondet B, et al Influence of spatial heterogeneity on an emerging infectious disease: the case of dengue epidemics. Proc Biol Sci. 2005;272: 1171–1177. 10.1098/rspb.2004.3020 16024379 PMC1559809

[84] Delatte H, Desvars A, Bouétard A, Bord S, Gimonneau G, Vourc’h G, et al Blood-feeding behavior of *Aedes albopictus*, a vector of Chikungunya on La Réunion. Vector Borne Zoonotic Dis. 2010;10: 249–258. 10.1089/vbz.2009.0026 19589060

[85] Delatte H, Gimonneau G, Triboire A, Fontenille D. Influence of temperature on immature development, survival, longevity, fecundity, and gonotrophic cycles of *Aedes albopictus*, vector of chikungunya and dengue in the Indian Ocean. J Med Entomol. 2009;46: 33–41. 10.1603/033.046.0105 19198515

[86] Microsoft, Weston S. foreach: Provides Foreach Looping Construct. Available: https://CRAN.R-project.org/package=foreach

[87] Microsoft Corporation, Weston S. doParallel: Foreach Parallel Adaptor for the “parallel” Package. 2019. Available: https://CRAN.R-project.org/package=doParallel

