# Supplementary figures and images for: Modeling intra-mosquito dynamics of Zika virus and its dose-dependence confirms the low epidemic potential of Aedes albopictus
Source: PLoS Pathog. 2020 Dec 31;16(12):e1009068. doi: 10.1371/journal.ppat.1009068 (PMC7774846; doi:10.1371/journal.ppat.1009068)

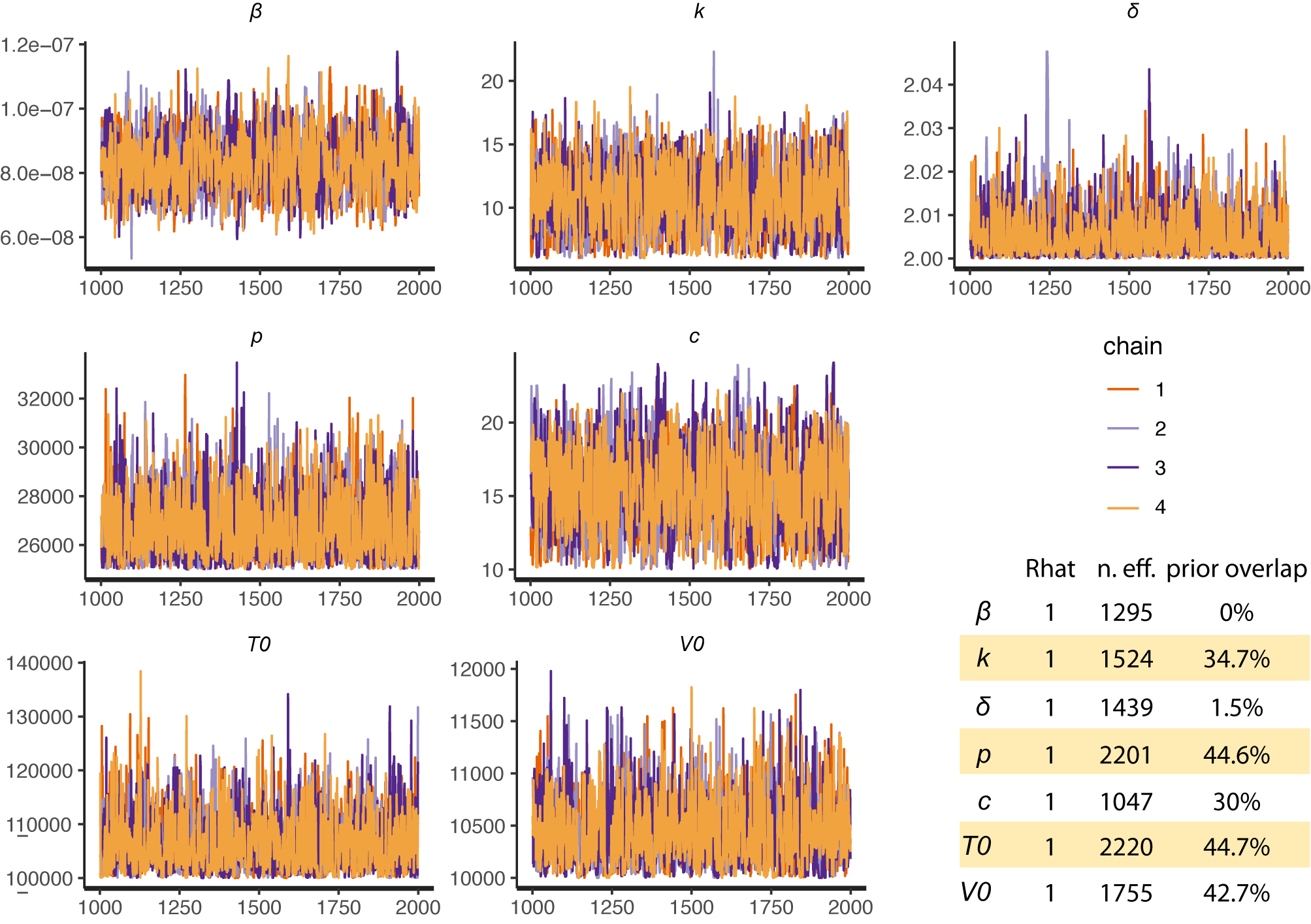

Supplement: S1 Fig — Trace plots of Markov chain Monte Carlo (MCMC) output for each of the 7 model parameters, with T0: the initial uninfected target cells concentration (cells per mL), V0: initial concentration of free virus particles (ZIKV RNA copies/mL), β: the infection rate constant of target cells per virion (per mL per day), k: rate of cell transition from a non-productive sate to a virus productive state (per day), δ: dying rate of productively infected cells (per day), p: free virus releasing rate by productively infected cells (per day) and c: virus clearance rate (per day). The model was run on 4 independent chains. The Rhat statistic, the number of effective samples, and the prior posterior overlap (PPO in %) are represented for each parameter. (TIF) [file ppat.1009068.s001.tif]

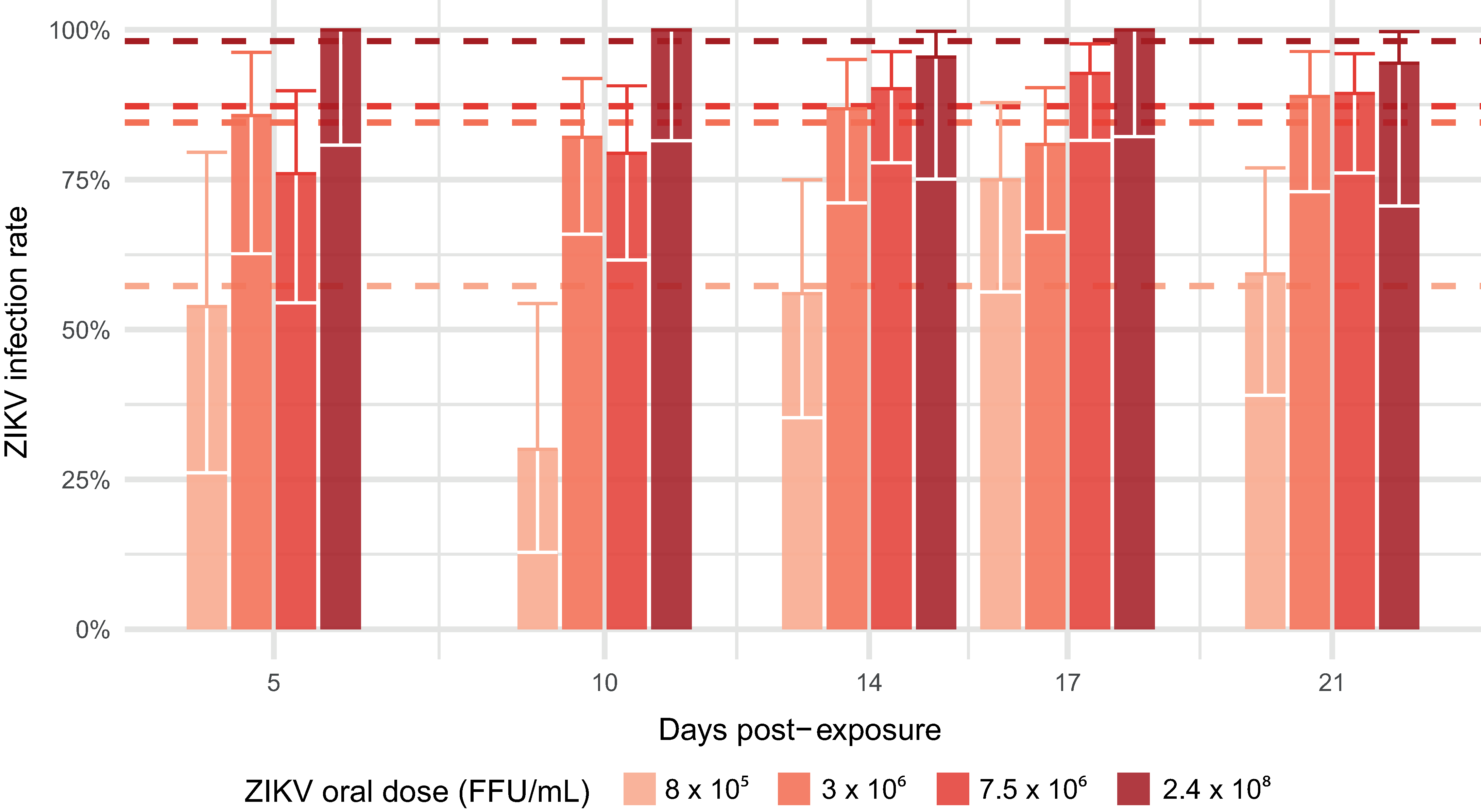

Supplement: S2 Fig — Percentages of body infections over time post-ZIKV exposure are represented at 4 infectious blood meal titer: 8 × 10^5^ FFU/mL, 3 × 10^6^ FFU/mL, 7.5 × 10^6^ FFU/mL and 2.4 × 10^8^ FFU/mL. 95% confidence intervals are indicated with the error bars, and prevalences averaged across time points are represented with a dashed line for each virus dose. (TIF) [file ppat.1009068.s002.tif]
