# Supplementary material for: Modeling intra-mosquito dynamics of Zika virus and its dose-dependence confirms the low epidemic potential of Aedes albopictus
Source: PLoS Pathog. 2020 Dec 31;16(12):e1009068. doi: 10.1371/journal.ppat.1009068 (PMC7774846; doi:10.1371/journal.ppat.1009068)
Supplement: Attachment — Submitted filename: PPATHOGENS-D-20-00924_Answer to reviewers.docx [file ppat.1009068.s008.docx]

**Manuscript -** Ref. No.:  PPATHOGENS-D-20-00924

**Title:** Modeling intra-mosquito dynamics of Zika virus and its dose-dependence confirms the low epidemic potential of *Aedes albopictus*

**Corresponding author:**

Albin Fontaine, Address: Institut de Recherche Biomédicale des Armées, Département des Maladies Infectieuses, Unité Parasitologie et Entomologie, IHU - Méditerranée Infection, 19-21 Boulevard Jean Moulin, 13385 Marseille Cedex 05, E-mail: [albinfont@gmail.com](mailto:albinfont@gmail.com)

Dear Editorial-Board Member,

According to Editorial-board and Reviewer comments, we hereby submit the revisions of our manuscript newly entitled “Modeling intra-mosquito dynamics of Zika virus and its dose-dependence confirms the low epidemic potential of *Aedes albopictus*”. We hope that our modifications and responses to the reviewers will correspond to the Editorial-board and reviewer expectations and follow the *PLOS Pathogens* journal guidelines. Changes were included in our manuscript with track changes as a word document and are also indicated in the cover letter (see below). A definitive version without track changes is also provided as requested.

Sincerely Yours,

Albin Fontaine

We would like to first acknowledge our reviewers sincerely for their time and expertise to this manuscript and for bringing constructive comments.

**Part II – Major Issues: Key Experiments Required for Acceptance**

1) **Reviewer #1: Critically, others have reported that *Aedes albopictus* are more susceptible to ZIKV infection than *Aedes aegypti*, but have a reduced transmission capacity, indicative of a transmission barrier. Unfortunately, since saliva was not collected and screened here, it is not possible to understand if this trend holds true with geographically distinct populations of *Aedes albopictus*. As a result, it is inaccurate throughout to say that vector competence was measured because transmission potential was not assessed. This should be explicitly stated along with the limitations.**

**Answer:** Virus detection in mosquito saliva is the gold standard for exploring vector competence because it directly assesses the transmission potential of a mosquito/pathogen couple. However, current forced salivation methods are prone to false negative errors that are poorly explored and/or acknowledged. False negativity can arise for two main reasons:

(1) There is no proper way to control that the mosquito salivated, especially when the saliva is collected in a non-oil-based fluid. Even when collected in oil, the saliva drop can be difficult to visualize.
(2) The amount of virus in the saliva is usually small, which can lead to an absence of detection due to losses during filtration/surfaces when working with small volumes (Garcia-Luna S.M. et al., 2018, <https://doi.org/10.1371/journal.pntd.0006599>).

Aside from these technical shortcomings, there are to our knowledge no formal proof that forced salivation assays are representative of “natural” salivation during a blood feeding (quality, volume, etc.), which can further lead to false negative issues. At last, scoring transmission event in single mosquitoes for our large cohort (625 mosquitoes in total) at different time points would have represented a logistical challenge. Based on our experience, we often encountered issues to model the dynamic of arbovirus transmission using our method because transmission rates over time often display an unexpected saw-tooth profile (instead of the expected S-shape profile) that might be linked to this false negative issue.

Systemic infection (*i.e.* dissemination) is a pre-requisite for the presence of virus in the saliva. Vector competence has been previously expressed in terms of dissemination rate, that is the percentage of engorged females with virus detected in the head (as a proxy for the salivary glands, which are located at the base of the mosquito head) (from the review of Souza-Neto et al. 2019, <https://doi.org/10.1016/j.meegid.2018.11.009>). Here, systemic infection was considered as a convenient proxy for transmission in our Agent Based Model. It of course assumes a total absence of salivary gland barriers and may thus provide wrong estimates of the underlying ‘true’ vector competence (overestimation proportion of the overall infectious mosquitoes) and/or EIP distribution (underestimation). It has however the advantage of not falling into type II errors.

We thus acknowledge that the presence of a transmission barrier would further scale down our predictions concerning the epidemic potential of *Ae. albopictus* for ZIKV. That was clearly stated in our discussion section. However, we believed that we succeeded to bring forward the dependency of intra-mosquito ZIKV infection and systemic infection dynamic on the virus dose and to further provide a model that allow to assess the relative epidemiological importance of parameters (such as mosquito densities). If reliable transmission assays are developed in the future, our ABM can be directly updated to consider these new data.

We acknowledge that the previous version of the manuscript did not discuss enough this fundamental limitation of our study. An emphasis is now made in the discussion section, as suggested by the reviewer. At last, the wording “vector competence” was replaced by infection rate or dissemination rate when possible, including in the abstract.

Lines 388-392 : “Variation of the transmission barrier permeability as a function of the virus dose or mosquito population was not addressed in our work*.* The presence of a transmission barrier would further scale down our predictions concerning the epidemic potential of Ae. albopictus for ZIKV but would not impact our main conclusions regarding the dependency of intra-mosquito ZIKV infection and systemic infection dynamic on the virus dose.”

2) **Line 114-116: vector competence, as a component of vectorial capacity, is governed by intrinsic factors that influence the ability of a mosquito species to vector a pathogen and is not interchangeable with the concept of vectorial capacity. Therefore, the time post-exposure when a mosquito becomes infectious—or the extrinsic incubation period—is a key factor that influences vectorial capacity, not vector competence.**

**Answer:** The original “Ross-Macdonald model” formula of vectorial capacity assumed that viremic human hosts are always infectious for the mosquito, but the formula has also been modified to include vector competence. In this vectorial capacity mathematical equation, vector competence is used as a time-independent proportion that acts as a limiting factor in the estimation of the mean number of potentially infective bites per person per day. ‘Vector competence’ is in this case viewed as the intrinsic ability of a mosquito species or population to transmit a pathogen. It can be measured experimentally as a ratio of mosquitoes that would ultimately transmit a pathogen over exposed mosquitoes (see for example the review from Souza-Neto et al. 2019, <https://doi.org/10.1016/j.meegid.2018.11.009>). But vector competence is also known to be influenced by extrinsic factors, such as temperature, or the infectious oral dose. Moreover, the transmitting mosquito ratio serving as a way to estimate vector competence is also strongly dependent of the time post pathogen exposure. By considering an experimental setting where a batch of mosquitoes would be orally exposed to a virus at day D0, all engorged mosquitoes would have a zero probability to have infectious virus in their saliva at this day (D0). The probability of virus transmission would then increase over time, as mosquitoes would incrementally reach their EIP, until to reach a plateau. At a population level, if one measure the same “infectious mosquito/exposed mosquitoes” ratio at 2 or 15 days after infection, he will not get the same result, hence not the same estimated ‘vector competence’. Most studies overlook that dynamical nature, and measure vector competence after a given time, considering that the “plateau” would be reached. We argue here, as others (e.g. Christofferson R.C., 2014, <https://doi.org/10.1371/journal.pone.0110538>), that considering the dynamic nature of ‘vector competence’ can be critical in the evaluation of vectorial capacity.

Vector competence can thus be considered as a complex dynamic variable that directly reflect the underlying distribution of the extrinsic incubation period (EIP) and to the proportion of mosquitoes that will never transmit the pathogen. One can transform empiric infectious/exposed mosquitoes ratio over time to obtain (1) the proportion of mosquitoes that will never transmit a pathogen, and (2) the distribution of EIP in the transmitting mosquito population, as it was performed in our manuscript. This time post virus exposure / ZIKV dose interaction was integrated in our Agent-Based Model (ABM). Unlike equation-based models, ABM has the ability to resolve complex interplay between parameters, including their interaction with time and can thus provide realistic vector-borne virus transmission frameworks that can be relatively easy to implement.

3) **Line 131: I believe that it would be useful to present the data for the estimate/establishment of physiologic human bloodmeal titers prior to describing any mosquito infection results. This is critical for understanding the physiologic relevance of your experimental vector competence assays.**

**Answer:** We agree with the reviewer, and the result section has been modified so that the “*Modeling of ZIKV loads dynamic in human venous blood*” part is now coming first. Figures numbers have been updated accordingly.

4) **Line 178: This section should explicitly state the temperature exposed mosquitoes were held at, because it has recently been reported that increased temperatures reduce ZIKV transmission rates in *Aedes albopictus* (PMID:31894724). Is 28C reflective of the climate in Marseilles and La Reunion?**

**Answer:** All our experiments were performed under a standardized 28°C temperature. Any consideration of a temperature effect would have dramatically complexify our experimental procedure (replication of the dose and population factor levels at all studied temperatures if we consider a full-factorial design). For information, 28°C is consistent with a mean daylight temperature from July to September in Marseille and with the mean daylight temperature in La Reunion during the interseason periods (April-May and October-November). The fact that all experiments were conducted at 28°C is now specified in the result section.

Line 196: “Mosquitoes were held at a standardized 28°C temperature during the rearing and experimental procedure.”

5) **Line 256: How is mosquito population density factored into the model described here? And if population density was factored in, is it representative of the overall population density of *Aedes albopictus* present in Marseille and/or La Reunion?**

**Answer:** Details on the epidemiological agent-based model parameters were missing in the initial version of the manuscript. To address this shortcoming, we have expended our Material and Methods section dedicated to the Agent-Based Model. Very briefly, mosquito density was approximated by the average number of bites each human experienced at every time step (a day). To account for daily heterogeneity, the number of bites each human agent experiences is drawn from a Poisson distribution of a mean centered on empirically set values (1, 5, 10 and 60). Our goal here was more to assess how individual mosquito biting load is influencing the initiation probability and magnitude of a ZIKV outbreak, rather than provide reliable forecast of the risk of ZIKV emergence in both places. We believe that our range of values captures the realistic variation of individual bites densities that can occur in both places over the year. Based on the paper of Delatte H. *et al*, 2010 (<https://doi.org/10.1089/vbz.2009.0026>), sixty bites per person per day is relevant to outdoor *Ae. albopictus* females biting activity on La Réunion island and might be considered as a high estimate.

Line 267: “The model was run 100 independent times over 365 days over a range of mean individual mosquito biting rates: 1, 5, 10 and 60 independent mosquitoes biting per person per day.”

Line 659-662: “Each human agent was exposed to a number of biting load drawn from a Poisson distribution with a mean value empirically set to 1, 5, 10 or 60 individual mosquito bites per person per day. Sixty bites per person per day is relevant to outdoor Ae. albopictus females biting activity on La Réunion island [86].”.

6) **Line 263: How was the EIP parameterized? This was not measured in your experimental system. It also is not clear how the daily probability of a host being fed upon and daily survival were factored in or estimated. Overall, using these data to estimate the overall vectorial capacity of the two populations of mosquitoes would be worthwhile.**

**Answer:** As detailed in answer #2, the EIP (or its systemic infection proxy based on virus detection in the mosquito’s head) was not directly measured but was inferred from the cumulative proportions of mosquitoes orally exposed to the virus with a systemic infection over time. These empirical data allows us to obtain (1) the proportion of mosquitoes that will never transmit, and (2) the distribution of “EIP” (time to systemic infection, a proxy for EIP) in the ultimately infectious mosquito population. We thus obtain the distribution of individual mosquito EIP values for the populations and the doses under consideration. These EIP distribution were modeled and then used in our ABM. We acknowledge that our EIP estimate is a convenient surrogate, and there is a probable lag time between dissemination and transmission. This lag time was not implemented in our system because it is difficult to estimate experimentally, for reasons listed in answer #1. It is however an approximation that standardized across all our conditions and do not impact our main conclusions.

The daily probably to get fed upon is related to answer #5 above: the number of mosquito that feed is drawn from a Poisson distribution (thus including 0) with the mean value being arbitrarily set to 1, 5, 10 or 60 individual mosquito bites per day. Daily survival was set to an arbitrary value of 0.85. Field data for daily survival probability of *Aedes albopictus* in Marseille or La Réunion are crucially missing, so we settled for a conservative estimate based on *Aedes aegypti* survival rates. More complete information about *nosoi* (the name of our ABM) can be found here: <https://doi.org/10.1111/2041-210X.13422> and <https://slequime.github.io/nosoi/>. We have added in the manuscript more details over some of the shared parameters in our model.

Lines 653-656: “It was assumed that human agents do not die from the infection and leave the simulation when they cure their infection after 12 days. Mosquitoes had a daily probability to die empirically set at 0.15 as used previously [43,85].”

7) **Line 345-348: here it appears that you are arguing that *Aedes albopictus* has a low epidemic potential because it might take upwards of 21 days for them to transmit virus. There are numerous examples of vector-pathogen relationships for which old-aged mosquitoes are the drivers of transmission and can successfully sustain epidemics. It would be worthwhile to discuss other factors that might make *Aedes albopictus* less likely to drive epidemics compared to *Aedes aegypti*, for example. In a previous section you allude to the fact that *Aedes albopictus* is more catholic in their feeding preferences. This could have an important role in their ability to maintain epidemic transmission.**

**Answer:** Old infected mosquitoes would always be at higher risk to transmit a pathogen than young ones because age is associated with a higher probability to survive through the extrinsic incubation period (EIP). However, for a constant given daily survival rate, the probability to survive through the EIP would increase as the duration of the EIP decreases, and vice-versa. Our results show that ZIKV had a relatively slow intra-host systemic infection dynamics when compared to other mosquito-borne viruses: by comparison, 100% systemic infections of *Ae. aegypti* can be observed for several dengue isolates at doses ranging from 5.7 to 5.85 log10 FFU/mL around 10 days (Fontaine A. et al., 2018, <https://doi.org/10.1371/journal.ppat.1007187> ), which is about 2.5 folds faster than for ZIKV. We showed that slow within-host dynamics is not an obstacle to successful transmission or outbreak initiation in presence of a high mosquito density (refer to answer (#8)).We agree with the reviewer that other factors might impact *Aedes albopictus* vector capacity. Host feeding preference, overall biting activity, seasonal dynamics, etc. would have an important impact on vector capacity. We however feel uncomfortable to discuss extensively these elements because none were explored in our study. In addition, host feeding preference would ultimately impact biting density that was assessed as a range in our study (see answer #6).

8) **Line 365: low compared to what? There are several examples of mosquitoes with low competence but high population density that have been capable of sustaining arbovirus outbreaks, e.g. YFV.**

**Answer:** The reviewer might refer to the paper of Miller *et al*. 1989, *Epidemic yellow fever caused by an incompetent mosquito vector*. Tropical Medicine and Parasitology, 40,396-399.
We agree with this comment and it is totally making our point. High mosquito population density can override a “low vector competence” of a vector for a pathogen. The conclusion of the Miller *et al*. 1989 study has been integrated to the discussion.

Lines 365-4369: “It has been previously demonstrated that an urban, *Aedes aegypti*-borne, epidemic of yellow fever occurred in 1987 in Africa although the mosquito vector was relatively resistant to infection and transmitted the virus inefficiently. It was suggested that a high mosquito density can override a relatively low vector competence to sustain active virus transmissions [52].”

9) **Line 379-393: given all of the caveats associated with ZIKV viremia in humans, would it be possible to, for example, use data on dengue virus—for which there is an extensive literature on viremia in humans—to demonstrate the accuracy of your predictions?**

**Answer:** Data on the literature on arbovirus load in human patients before symptom onsets is not so extensive, including for dengue virus. This is due to the difficulty to detect infected cases during the pre-symptomatic stage of the disease. Duong V. and colleagues (*Asymptomatic humans transmit dengue virus to mosquitoes*, 2015, PNAS, <https://doi.org/10.1073/pnas.1508114112>) succeeded to collect dengue virus (DENV) loads in patient plasma before symptom onsets based on geographic cluster sampling around index cases. These data were partially used to model DENV loads dynamics in human plasma by Quirine A. ten Bosch et al. *Contributions from the silent majority dominate dengue virus transmission*, 2018, PLoS Pathog. <https://doi.org/10.1371/journal.ppat.1006965>) using a similar approach to ours (*i.e*. fitting ordinary differential equations that describe within-human host plasma viral load dynamic with a Bayesian approach). Different models and initial parameter values have been used in this study and ours, which hinders direct comparison. It can however be noted that our within-human host plasma viral load dynamic prediction for ZIKV matched relatively well dynamic obtained with DENV in term of magnitude (*i.e*. around 8 log_10_ copies/mL at the peak of viremia) but less in term of timeliness (higher time to reach both the peak of virus load and virus fading for DENV as compared to our ZIKV predictions, but that might at least be partially explained by a lower initial virus inoculum). Instead of mixing human viremia data from different virus species, we made the choice to only use ZIKV viremia data from primates (which include human). We inferred model parameters using non-human primate viremia dynamics as strong priors in our model fitting process on human data under the strong hypothesis that human and non-human viremia dynamics are relatively similar. Our aim was to produce a coherent intra-human virus dynamic to assess how it can influence intra-mosquito infection probability and systemic infection dynamic. Any deviation from this hypothesis would impact our predictions, we believe to have clearly specified this caveat in our manuscript.

10) **Reviewer #2: Methods regarding human viral loads: It is not immediately clear how ZIKV was quantified using RT-PCR. As described (lines 561-581), there is no probe or lightcycler so how is a Cq value derived? Was the human data already published, this was not very clear as the methods makes it seem as this information is primary data.**

**Answer:** We apologize for the confusion. The “ZIKV RNA detection” (lines 561-581) section uniquely concerns the molecular procedure that has been used to detect ZIKV in mosquitoes. This has been done qualitatively (presence/absence) by revealing an amplified target of the virus genomic RNA on a gel.

We used ZIKV viremia data (in kinetic) from venous serum samples of 35 patients. Virus load data from 21 of these patients were already published (Matheus S. *et al*. *Zika Virus Persistence and Higher Viral Loads in Cutaneous Capillaries Than in Venous Blood.* Emerging Infect Dis. 2017, <https://doi.org/10.3201/eid2311.170337>). Thirteen additional patients from whom sera were only collected from venous blood were added. These patients came from a follow-up study that assessed ZIKV symptom duration and quality of life post symptoms (de Laval F, *et al*. *Evolution of symptoms and quality of life during Zika virus infection: A 1-year prospective cohort study*. J Clin Virol. 2018;109: 57–62. <https://doi.org/10.1016/j.jcv.2018.09.015>). The same procedure was applied in both studies to detect and quantify ZIKV loads in venous blood (*i.e*. RealStar Zika Virus real-time reverse-transcription PCR (RT-PCR) Kit 1.0 (Altona Diagnostics GmbH, Hamburg, Germany). Virus loads were not published for this second paper and can be thus considered as primary data here. This was added in the material and methods section.

Line 435: “Thirteen additional patients from whom sera were only collected from venous blood were added in this study (virus loads for these patients can be considered here as primary data) [64].”

11) **Line 487: Studies have demonstrated that serial passage in C6/36 cells can attenuate the virus in mosquitoes. Was a comparison made to virus passaged at least once through a mammalian cell line?**

**Answer:** The requirement of arboviruses to replicate in both vertebrate and arthropod hosts, each of which imposes specific selective pressures, is considered to be a key determinant in their genetic stability. We are not sure to understand what the term attenuation is referred to by the reviewer. Serial passages in insect cells (such as C6/36 *Ae. albopictus* cells) can lead to a virus adaptation for optimal fitness in the insect host. However, these adaptations usually occur after numerous serial passages (for instance: > 30 serial passages based on Ali S.M. et al., 2018, <https://doi.org/10.1038/s41598-018-34561-x>; 10 serial passages in the work of Coffey L.L et al., 2008, <https://doi.org/10.1073/pnas.0712130105>). Our virus isolate was passaged only four times in C6/36 cells, which were reported to have little effect on virus evolution (Coffey L.L et al., 2008, <https://doi.org/10.1073/pnas.0712130105>, Brackney, D. E. et al. 2010, <https://doi.org/10.1371/journal.pntd.0000856>). We did not consider the isolate variability effect on ZIKV infection probability and systemic infection dynamics in our study. This can be considered as an interesting perspective. We selected a ZIKV isolate with a relatively low passage history that is representative of the ZIKV isolates that have emerged in the Americas. We believe that small virus adaptation to insect cells or virus genomic mutation, if any, would not have hindered our main conclusions, and would have been shared across the different experimental conditions.

12) **Line 536: 9-13 days post emergence is quite old for a vector competence study. What is the justification for this?**

**Answer:** Preliminary blood-feeding experiments (unpublished) have revealed that time post-emergence was a key determinant of blood-engorgement rates in our laboratory settings, younger mosquitoes failing to engorge at a high enough rate (< 50%). Achieving high blood-feeding rates upon experimental ZIKV oral exposure was a prerequisite to obtain sufficient sample sizes to score intra-mosquito virus dynamics. The within-mosquito dynamics of vector competence/EIP were reported to be not significant among treatments where mosquitoes were exposed at different ages (Mayton H.E., et al. 2020, <https://doi.org/10.1186/s13071-020-04181-4>). We therefore do not expect any obvious bias due to the use of slightly older mosquitoes.

13) **Overall: The focus of the paper seems to be more about the dose-dependence leading to this within-vector heterogeneity. The title and conclusions could be better stated to match what the experimental design and structure of the results suggest is the premise of the study.**

**Answer:** The focus of the paper was indeed to consider the impact of the infectious dose on the within-mosquito infection dynamics. But moreover, it aimed to consider two highly overlooked factors, time-dependence of ‘vector competence’ (referred here as systemic infection) (see answer #2) and dose, into an approximated estimation of the vectorial capacity of *Aedes albopictus* for ZIKV. We propose to change our title to: “Modeling intra-mosquito dynamics of Zika virus and its dose-dependence confirms the low epidemic potential of *Aedes albopictus*”. We hope that the current version of the discussion will better match the premise of the study.

**Part III – Minor Issues: Editorial and Data Presentation Modifications**

14) **Reviewer #1: Line 34-38 are inaccurate. *Aedes albopictus* was implicated as the vector of a Zika virus outbreak in Gabon in 2007: PMID: 24516683.**

**Answer:** The implication of *Ae. albopictus* as the vector of ZIKV in Gabon in 2007-2010 was strongly suspected but not fully demonstrated. The authors conducted a retrospective study of human sera and mosquitoes collected in Gabon from 2007 to 2010, to identify other arboviruses than chikungunya and dengue viruses. They retrospectively identified the virus in 5 human sera and 2 *Aedes albopictus* pools. Because *Ae. albopictus* was a vector in the chikungunya and dengue viruses outbreaks, its role in transmitting ZIKV in human was strongly suspected. However, the main vector of ZIKV, *Ae. aegypti*, was also present on the study sites. We specified in the introduction section that *Ae. albopictus* was strongly suspected in ZIKV transmission in Central Africa and cited this work. Our sentence was modified as:

Line 33-36: “Although *Aedes albopictus* can transmit ZIKV experimentally and was suspected to be a ZIKV vector in Central Africa, the potential of this species to sustain virus transmission was yet to be uncovered until the end of 2019, when several autochthonous transmissions of the virus vectored by *Ae. albopictus* occurred in France.”

15) **Line 77-80: The statements here are confusing. There are other mosquito species that vector pathogens of public health concern.**

**Answer:** We totally agree with the fact that other mosquito species can transmit pathogens of public health concern. However, *Aedes aegypti* and *Aedes albopictus* can be considered as the main vectors of viruses of human health importance, in term of incidence and mortality. The sentence was modified to avoid confusion.

Line 78-81: “While the yellow fever mosquito *Aedes aegypti* is considered as the primary vector of viruses affecting human health, the Asian tiger mosquito *Aedes albopictus* might progressively take the lead because of its outstanding invasive capacity [1] and its ability to transmit numerous virus species experimentally [2].”

16) **Line 81: “continents” should be “inhabited continents”—I don’t believe *Aedes albopictus* is present on Antarctica.**

**Answer:** This sentence was modified as suggested by the reviewer.

17) **Line 93: Given all of the emerging evidence, it is inaccurate to characterize ZIKV infection as “usually mild”.**

**Answer:** This sentence was modified as suggested by the reviewer.

Line 94-97: “Most ZIKV infections are asymptomatic or cause self-limiting febrile sickness but are sometimes associated with complications, including Guillain-Barré syndrome or congenital microcephaly or fetal losses in women infected during pregnancy [14].”

18) **Line 102-104: The references cited do not directly compare ZIKV infection, dissemination, and transmission rates in *Aedes aegypti* and/or *Aedes albopictus* to those of the same mosquitoes exposed to DENV, CHIKV, or YFV (and one is a meta analysis), so is therefore a misrepresentation of the available data.**

**Answer:** We agree with this comment. No solid conclusions can be drawn if vector competence was not compared across virus species in standardized conditions. Our objective was to highlight the fact that several studies had conclude about a limited risk of ZIKV transmission in areas with *Ae. albopictus* as a unique vector, due to the observed low dissemination and transmission rates. This part was modified:

Line 104: “The presence of barriers preventing systemic ZIKV infection (virus dissemination from the midgut to secondary tissues) or transmission were repeatedly reported for *Ae. albopictus* [22,24–27] and suggested a limited risk of Zika virus transmission in Europe or La Réunion island [22,25].”

19) **Line 134: please list the geographic origin of the ZIKV isolate used.**

**Answer:** The geographic origin of the ZIKV isolate was added in this result section.

Line 164: “The virus was isolated from the plasma of a traveler returning from Suriname in 2016 that belongs to the Asian lineage.”

20) **Line 142-143: what was the time point for these infection prevalence results?**

**Answer:** These prevalence results were averaged over time as describe in the sentence and are described as mean prevalences.

21) **Line 195: this section should be presented first in the Results.**

**Answer:** The result section has been modified so that the “Modeling of ZIKV loads dynamic in human venous blood” part is coming first, as suggested by both reviewers (refer to comment 3).

22) **Line 213-214: How did you establish the PFU:particle ratio here?**

**Answer:** Predicted ZIKV RNA loads (copies/mL) were converted into infectious virus loads (FFU/mL) by dividing the viremia predicted by the model by a fixed arbitrary value of 750. This value lies at the midrange of the RNA copies/PFU ratio estimated on a French Polynesian ZIKV isolate (Dudley et al., 2016). Infectious titers were reported to be 500–1,000-fold less than ZIKV RNA copies concentrations in non-human primate plasma (Dudley et al., 2016, <https://doi.org/10.1038/ncomms12204>).

This was also considered in the ABM model, as the human-within host dynamics model models RNA copies, but our dose-response model is in PFU. To acknowledge for heterogeneity, the value for the ratio was sampled randomly for each human upon transmission to a mosquito in a uniform distribution between 500 and 1,000.

These indications are now clearly stated in the material and method section:

Lines 511-515: “Predicted ZIKV RNA loads (copies/mL) were converted into infectious virus loads (FFU/mL) by dividing the viremia predicted by the model by a fixed arbitrary value of 750. This value lies at the midrange of the RNA copies/PFU ratios (infectious titers were reported to be 500–1,000-fold less than ZIKV RNA copies concentrations) that were estimated in non-human primate plasma with a French Polynesian ZIKV isolate [73].”

23) **Line 310-312: other previous studies have considered the inter-relationship between both within host dynamics.**

**Answer:** As far as we are aware, most previous works that have addressed the inter-relationship between both within-host dynamics used modelling approach without empirical data (*e.g.* Althouse and Hanley, 2015, <https://doi.org/10.1098/rstb.2014.0299>, or Lord et al., 2006, [https://doi.org/10.1603/0022-2585(2006)43[623:rbhvav]2.0.co;2](https://doi.org/10.1603/0022-2585(2006)43%5b623:rbhvav%5d2.0.co;2)). One paper is indeed explicitly exploring the ZIKV dose effect on infection and dissemination dynamic in the *Aedes aegypti* mosquito model (Tesla B. et al., 2018, <https://journals.plos.org/plosntds/article?id=10.1371/journal.pntd.0006733>). The authors empirically demonstrated the dependency of infection rate, systemic infection, and transmission dynamic on ZIKV oral viral dose in *Ae. aegypti*. Our results are now compared with the results of this paper in our discussion section. We still believe that the consideration of the inter-relationship between both within-hosts dynamics involved in the virus life cycle coupled with empirical data for both humans and mosquitoes, is a major originality of our study. If the reviewer has other papers in mind, we will welcome suggestions.

Lines 352-364: “Tesla B. and colleagues have previously empirically demonstrated the dependency of infection rate, systemic infection and transmission dynamic on ZIKV oral viral dose using the *Ae. aegypti* mosquito model [51]. They estimated at 4.98 log10 Plaque Forming Units (PFU)/mL the infectious dose required to infect 50% of the mosquito population (ID50). By applying our analysis procedure (Supplementary file 4) on their raw data, we estimated the time estimates to reach 50% systemic infection to be 18.7, 14 and 8.2 days across their 3 replicates for a 5 log10 PFU/mL oral dose. By comparison, we estimated in *Ae. albopictus* the ID50 at 5.6 log10 FFU/mL and at 19.2 days the median systemic infection estimate for a similar ZIKV oral dose (5 log10 FFU/mL). These results suggest the *Ae. aegypti* mosquito species vector competence for ZIKV is not fundamentally superior than Ae. albopictus, as it has been previously reported [37].”

24) **Line 314: Other studies have demonstrated a dose-dependent relationship between bloodmeal titer and *Aedes albopictus* infection rates. Those should be discussed and your study placed in context with what has been demonstrated previously.**

**Answer:** In the same paragraph from line 318, we specifically compared our ID50 with two published studies that have assessed *Ae. albopictus* infection rates as a function of the ZIKV dose. *“To our knowledge, only two studies have assessed Ae. albopictus infection rates as a function of the ZIKV dose [26,27]. The oral median infection dose (ID50) estimated by these studies ranged from 6.1 to 6.8 log10 FFU/mL. Our ID50 estimate (5.6 log10 FFU/mL (95% CI: 5.3 log10 FFU/m 321 L - 5.8 log10 FFU/mL)) was in the same order of magnitude (…).”.* Tesla B. et al. 2018 (refer to comment 23) worked on *Ae. aegypti* and estimated at 4.98 log10 PFU/mL the oral median infection dose (ID50). That reference was added in the discussion section. We would be happy to add any other study the reviewer has in mind.

25) **Line 356: *Aedes albopictus* is notorious for taking multiple, intermittent bloodmeals, and bloodmeals while still carrying eggs, so a daily bite rate of 1 may not be biologically accurate and higher bite rates may be more in line with what happens in nature. In sum, it is not clear to this reviewer whether this was factored into this statement in the manuscript.**

**Answer:** We agree with this comment. It is not straightforward to estimate a reliable daily biting rate estimate as it can change in time and at a very local scale. Therefore, we actually based our simulation study on a range of biting loads, with mean values ranging from 1 bite to 60 bites a day per humans (see our answer #5). On the mosquito side, bites are only considered for their transmission aspect, and a mosquito, when biting that day, would only transmit to one human. To our knowledge, there is a critical lack of empirical data to assess if *Aedes albopictus* can 100% transmit the virus when biting several times once infectious. Implication of this point in an epidemiological context remains to be explored into more details. This aspect was not considered in our manuscript because of the lack of data. However, our ABM can incorporate such data should they be available.

26) **Line 484: was your ZIKV isolate used in your vector competence studies sequence confirmed and what if any reported differences were there between it and the GenBank sequence?**

**Answer:** We had access to our ZIKV isolate through the European Virus Archive - global (EVAg) portal. Our virus isolate was passaged only four times in C6/36 cells, which were reported to have marginal effect on virus evolution (Coffey L.L et al., 2008, <https://doi.org/10.1073/pnas.0712130105>, Brackney, D. E. et al. 2010, <https://doi.org/10.1371/journal.pntd.0000856>) (refer to comment 11). We did not re-sequence our isolate genomic RNA to confirm its consensus sequence. Assessing any variation effect on vector competence across ZIKV isolates was beyond the scope of our study. Any other ZIKV isolates could have been used as a surrogate here.

27) **Figure 1: It is difficult to distinguish the size of the dots that represent sample size. It also is not immediately clear what the difference is between light and dark red and blue. And I confess it took me a few minutes of searching to realize the timepoint was listed only in panels C and D.**

**Answer:** The figure was modified to include the time post-virus exposure in all panels. Sample size is represented with a dot size scale and we believe that it would overload the figure to write number below each dot. We do not see any obvious way to improve this point without impacting the figure clarity. In addition, sample sizes are represented for information purpose and are not mandatory for the figure main message. If needed, sample size can be easily accessed on Supplementary file 3. A clear separation was now added between panels A, C and B, D to make clearer that they represent two different doses.

28) **Figure 2: There are only 3 dots on the graph but 4 dots in the legend. I suggest making the legend reflect what is shown on the graph.**

**Answer:** There are 4 dots on the graph, one is indeed small. The dot size on the legend is provided as a scale and not as a discrete value. Sample size are now further indicated on the figure with numbers.

29) **Reviewer #2: Please indicate the type of model used in the abstract.**

**Answer:** It is now indicated in the abstract that an Agent based model was used to perform the epidemiological simulations.

Lines 44: “(…) and (iii) in silico epidemiological simulations using an Agent-Based Model.”

30) **Reference needed for sentence at Lines 114-116 (The intrinsic ability of…)**

**Answer:** The “Tesla B, Demakovsky LR, Packiam HS, et al. 2018, Estimating the effects of variation in viremia on mosquito susceptibility, infectiousness, and R0 of Zika in *Aedes aegypti*. PLoS Negl Trop Dis. 2018;12(8):e0006733, PLoS neglected tropical diseases” reference was added to this sentence.

31) **Line 464: What is the range of these averaged medians? Model sensitivity to incubation periods has been demonstrated, and understanding the range of this parameter is important for inferring model robustness.**

**Answer:** Relatively restricted credible or confidence intervals around the mean were observed for all three studies with 5.9 days (95% credible interval, 4.4–7.6 days) for Lessler J. et al., 6.2 (95% CI 5.7––6.6) for Krow-Lucal E.R. et al. and 6.8 (95% CI, 5.8–7.7 days) for Fourié T. et al..

Lines 489-493: “This six-day incubation period corresponds to the rounded averaged median time of the ZIKV incubation period estimated from different independent studies (i.e. 5.9 days (95% credible interval, 4.4–7.6 days) for Lessler J. et al., 6.2 (95% CI 5.7––6.6) for Krow-Lucal E.R. et al., and 6.8 (95% CI, 5.8–7.7 days) for Fourié T. et al.) [67–69].”

32) **Line 645: References needed for systemic infection as a proxy for transmission since detection in saliva has become the norm for transmission estimation. This also needs to be clearly stated as a caveat of the paper and model in the Discussion.**

**Answer:** Despite currently being considered as the gold standard, detection of virus in saliva suffers several shortcomings, one of which is type II errors (false negative; see our response to comment #1). For that reason, detection of arboviruses infectious particles or RNA in mosquito head or leg tissues was used here as a convenient proxy, because dissemination is a necessary step for salivary gland infection. We acknowledge however that this assumes a lack of salivary gland infection and escape barrier and that the virus presence in mosquito head tissues indicated the potential for onward transmission. This may thus provide non accurate estimates of the *Aedes albopictus*’ vector competence for ZIKV, and thereby its vector capacity, which does not question our main conclusions. This limitation has now been, we believe, fully addressed in our discussion (see comment #1).
